# Multisystemic manifestations in a cohort of 75 classical Ehlers-Danlos syndrome patients: natural history and nosological perspectives

**DOI:** 10.1186/s13023-020-01470-0

**Published:** 2020-07-31

**Authors:** Marco Ritelli, Marina Venturini, Valeria Cinquina, Nicola Chiarelli, Marina Colombi

**Affiliations:** 1 Division of Biology and Genetics, Department of Molecular and Translational Medicine, University of Brescia, Viale Europa 11, I-25123 Brescia, Italy; 2 Division of Dermatology, Department of Clinical and Experimental Sciences, Spedali Civili University Hospital, Brescia, Italy

**Keywords:** Classical Ehlers-Danlos syndrome, Nosology, Atrophic scars, Skin hyperextensibility, Joint hypermobility, Multisystemic involvement, Natural history, *COL5A1*, *COL5A2*, *COL1A1*

## Abstract

**Background:**

The Ehlers-Danlos syndromes (EDS) are rare connective tissue disorders consisting of 13 subtypes with overlapping features including joint hypermobility, skin and generalized connective tissue fragility. Classical EDS (cEDS) is principally caused by heterozygous *COL5A1* or *COL5A2* variants and rarely by the *COL1A1* p.(Arg312Cys) substitution. Current major criteria are (1) skin hyperextensibility plus atrophic scars and (2) generalized joint hypermobility (gJHM). Minor criteria include additional mucocutaneous signs, epicanthal folds, gJHM complications, and an affected first-degree relative. Minimal criteria prompting molecular testing are major criterion 1 plus either major criterion 2 or 3 minor criteria. In addition to these features, the clinical picture also involves multiple organ systems, but large-scale cohort studies are still missing. This study aimed to investigate the multisystemic involvement and natural history of cEDS through a cross-sectional study on a cohort of 75 molecularly confirmed patients evaluated from 2010 to 2019 in a tertiary referral center. The diagnostic criteria, additional mucocutaneous, osteoarticular, musculoskeletal, cardiovascular, gastrointestinal, uro-gynecological, neuropsychiatric, and atopic issues, and facial/ocular features were ascertained, and feature rates compared by sex and age.

**Results:**

Our study confirms that cEDS is mainly characterized by cutaneous and articular involvement, though none of their hallmarks was represented in all cases and suggests a milder multisystemic involvement and a more favorable natural history compared to other EDS subtypes. Abnormal scarring was the most frequent and characteristic sign, skin hyperextensibility and gJHM were less common, all without any sex and age bias; joint instability complications were more recurrent in adults. Some orthopedic features showed a high prevalence, whereas the other issues related to the investigated organ systems were less recurrent with few exceptions and age-related differences.

**Conclusions:**

Our findings define the diagnostic relevance of cutaneous and articular features and additional clinical signs associated to cEDS. Furthermore, our data suggest an update of the current EDS nosology concerning scarring that should be considered separately from skin hyperextensibility and that the clinical diagnosis of cEDS may be enhanced by the accurate evaluation of orthopedic manifestations at all ages, faciocutaneous indicators in children, and some acquired traits related to joint instability complications, premature skin aging, and patterning of abnormal scarring in older individuals.

## Background

Ehlers–Danlos syndromes (EDS) represent a clinically and genetically heterogeneous group of heritable connective tissue disorders (HCTDs) sharing a variable combination of skin hyperextensibility, joint hypermobility (JHM), and internal organ and vessel fragility. The 2017 international classification of EDS recognizes 13 subtypes that are caused by pathogenic variants in 19 different genes, mainly encoding fibrillar collagens, collagen-modifying proteins, or processing enzymes [1]. The classical (cEDS), vascular (vEDS), and the molecularly unsolved hypermobile (hEDS) EDS subtypes account for more than 90% of patients. cEDS (MIM #130000) has an estimated prevalence of 1/20,000 and it is principally caused by heterozygous pathogenic variants in *COL5A1* or *COL5A2* encoding type V collagen and rarely by the c.934C > T p.(Arg312Cys) missense variant in *COL1A1* encoding type I collagen [1–14].

Although cEDS is recognized since the seventeenth century because of the remarkable cutaneous and articular involvement, it remains poorly defined on clinical grounds and its diagnosis is still based on experts’ opinion rather than systematic published data. According to the up-to-date EDS nosology, cEDS should be suspected in the simultaneous presence of skin hyperextensibility plus atrophic scarring (major criterion 1). This combined criterion must be present together with the other major criterion, i.e., generalized JHM (gJHM) assessed with the Beighton score (BS) [15], and/or with at least three of the following minor criteria: easy bruising, soft, doughy skin, skin fragility, molluscoid pseudotumors, subcutaneous spheroids, hernia (or a history of thereof), epicanthal folds, JHM complications (sprains, luxation/subluxation, pain, flexible flatfoot), and family history of a first degree relative who meets clinical criteria [1, 16]. Skin hyperextensibility is formally defined as the stretching of the skin over a standardized cut off in 3 of the following areas: 1.5 cm for the distal part of the forearms and the dorsum of the hands and 3 cm for neck, elbows, and knees [1, 7, 16, 17]. Although atrophic scarring can range in severity, most cEDS patients have very poor wound healing leading to multiple, widened atrophic scars in different body areas, especially over pressure points and areas prone to trauma [6, 7, 16]. In addition to the nosological criteria, the clinical picture of cEDS variably involves multiple organ systems, but observational data in large cohorts of molecularly proven cEDS patients are still limited. In particular, it is reported that cEDS patients may show delayed motor development with mild hypotonia, characteristic facial features, fatigue and muscle cramps, premature rupture of fetal membranes, cervical insufficiency during pregnancy, rectal prolapse in early childhood, mild scoliosis, and cardiac/blood vessel fragility including mitral/tricuspid valve prolapse, and aortic root dilatation. In very rare individuals, especially (but not exclusively) in those with the *COL1A1* p.(Arg312Cys) missense variant, spontaneous rupture of large arteries, intracranial aneurysms, and arteriovenous fistulae may occur [5–7, 9–14, 18–26].

A definite diagnosis of cEDS is established by the identification on molecular genetic testing of a heterozygous pathogenic variant in one of the cEDS-associated genes. The largest part of patients carry small *COL5A1* pathogenic variants and the majority of these are null alleles (nonsense, frameshift, out-of-frame splice) leading to functional haploinsufficiency; some missense variants, especially within the collagenous domain of the protein, and a few intragenic rearrangements including (multi) exon deletions/duplications are also described [6, 7]. In *COL5A2*, structural variants (missense, in-frame splice) exerting a dominant negative effect are the most common [4–6, 16]. In patients who fulfill the main clinical features of cEDS, the variant detection rate is about 90% [5, 6]. No clear-cut genotype-phenotype correlations have emerged so far, except that *COL5A2* variants seem to result in a more severe phenotype, although numbers are still limited. The EDS Leiden Open Variation Database (LOVD) [27] contains approximately 200 and 60 unique *COL5A1* and *COL5A2* pathogenic variants, respectively. Until recently, genetic testing was principally based on (serial) single-gene testing by Sanger sequencing, i.e., analysis of *COL5A1* is performed first, followed by *COL5A2* analysis and then gene-targeted copy number variant analysis to identify large deletions or duplications. If no pathogenic variant is found, the search for the recurrent *COL1A1* p.(Arg312Cys) variant should be performed [1, 6, 16]. As specific types of mutations may be lost due to technical limits, negative molecular testing does not exclude the diagnosis of cEDS; however, alternative diagnosis should be considered mainly if the patient’s phenotype is truly doubtful [1]. Indeed, recognition of cEDS is generally not challenging, since most patients present with the typical cutaneous hallmarks and gJHM (BS ≥5/9). However, vast intra- and interfamilial variability tells a much wider clinical presentation [4–6] and an important overlap with other EDS subtypes and HCTDs [1, 9, 16, 28–30]. Nowadays, molecular analysis, especially for doubtful patients, should rely on next generation sequencing (NGS) technologies, including multigene panels containing at least all EDS-associated genes and/or panels comprising genes of the major HCTDs in differential diagnosis and/or a custom phenotype-focused whole exome analysis [1]. Herein, we present a cross-sectional study on a cohort of 75 cEDS patients from 44 families with a confirmed molecular diagnosis focusing on the diagnostic criteria recognized in the current EDS nosology and on additional mucocutaneous, osteoarticular, musculoskeletal, cardiovascular, gastrointestinal, uro-gynecological, neuropsychiatric, atopic, and facial/ocular features, in order to investigate the multisystemic involvement and natural history of the disorder.

## Patients and methods

### Patients’ cohort and evaluated clinical features

Seventy-five patients, 44 index-cases and 31 relatives, with cEDS were evaluated from 2010 to 2019 in a tertiary referral center for the diagnosis and management of HCTDs (i.e., “Ehlers-Danlos Syndrome and Inherited Connective Tissue Disorders Outpatient Clinic (CESED)” at the Spedali Civili University Hospital of Brescia. Until February 2017, all clinical signs included in the Villefranche nosology [15] were evaluated prior to genetic testing. After the publication of the revised EDS classification, the new criteria were applied for confirmatory molecular analysis and clinical features of the previously evaluated patients were reanalyzed according to the novel guidelines. In addition, several previously diagnosed patients were reevaluated during follow-ups (for number and age at visits see Additional Table 1).

**Fig. 1:**
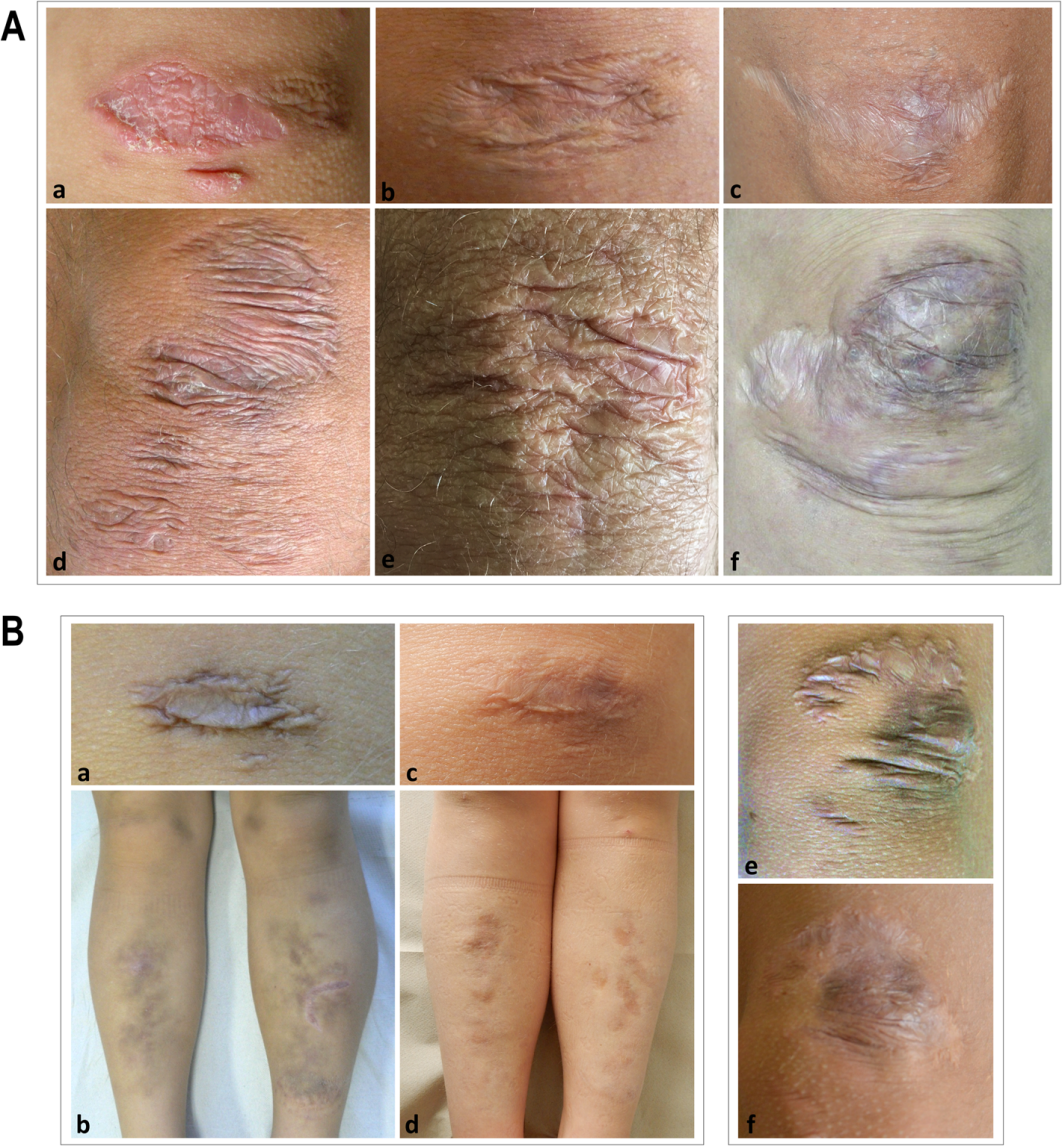
**A**) Representative images of different atrophic scars on cEDS patients’ knees. A healing scar in a 6-year-old girl (**a**); a papyraceous, non-hemosiderotic scar in a 22-year-old female (**b**); a cigarette paper scar in a 39-year-old female (**c**); multiple papyraceous, hemosiderotic scars in a 40-year-old female (**d**); multiple papyraceous scars in a 57-year-old male (**e**); combination of multiple, papyraceous, hemosiderotic scars, and cigarette paper scars in a 34-year-old female (**f**). **B**) Evolution of papyraceous, and hemosiderotic scars in 2 children assessed at increasing ages. On the left, a single scar on the knee and multiple scars at the pretibial area in a girl at 6 (**a, b**) and 11 years of age (**c, d**); on the right, multiple scars on the knee in a boy at 9 (**e**) and 12 years of age (**f**)

**Fig. 2:**
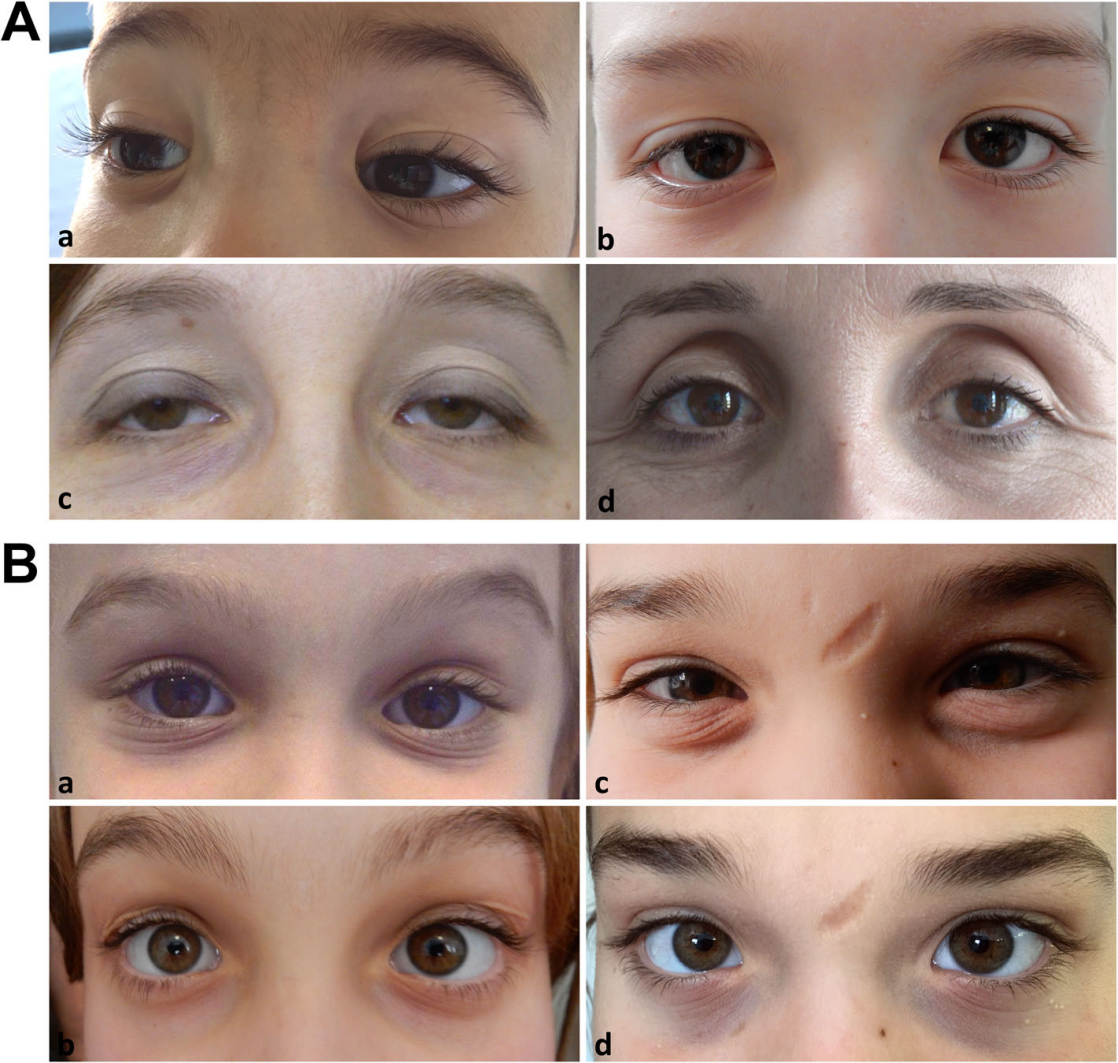
**A)** Representative images of characteristic ocular features in cEDS patients from different ages. Epicanthal folds in 2 boys at 3 (**a**) and 12 years of age (**b**); palpebral ptosis (**c**) and sunken eyes (**d**) in two over 30 years old females. **B**) Evolution of facial gestalt in 2 children assessed at increasing ages. On the left, infraorbital creases and bluish sclerae in a girl at 6 (**a**) and 11 years of age (**b**); on the right, infraorbital creases and facial scars in a girl at 10 (**c**) and 15 years of age (**d**)

Direct physical examination of available patients systematically consisted in different steps. First, facial features, including frontal and other facial scars, ocular anomalies and other traits, as well as the appearance, location, and number of scars all over the body were searched and documented by clinical photographs (Figs. 1 and 2). Ocular signs included epicanthal folds, palpebral ptosis, sunken eyes, infraorbital creases, hypotelorism, hypertelorism/telecanthus, myopia, strabismus, xeropthalmia, and elongated/up-slanting palpebral fissures (Fig. 2). Additional investigated facial features comprised micro/retrognathia, low-set ears, anteverted nostrils, elongated philtrum, and hypoplastic auricular lobe. Atrophic scars were extremely variable in clinical appearance; in particular, 3 different frequent morphologies were identified and classified as papyraceous, hemosiderotic, and cigarette paper (Fig. 1). Scarring was designed positive only in patients with multiple, widened scars at the typical areas (knees, elbows, forehead, shins, chin, etc.), whereas small, isolated, single, or post-surgical scars were not considered representative of cEDS. Second, to asses texture/consistence, the skin was touched at different sites including arms, thorax, hands and legs. Soft/velvety/doughy skin was an entirely subjective feeling developed during clinical practice. Third, to assess hyperextensibility, the skin was stretched in specific points comprising upper eyelids, neck, dorsum of hand and forearm, chest, abdomen, elbows, and knees. Skin was considered hyperextensible when overextended according to the standardized cut off values in at least 3 of the areas defined in the 2017 EDS nosology [1]. Further evaluated mucocutaneous features included molluscoid pseudotumors, easy bruising, subcutaneous spheroids, inguinal, umbilical, and incisional hernia, keloid formation, gingival inflammation/recession, livedo reticularis, keratosis pilaris/hyperkeratosis of extensor surfaces, xerosis, striae distensae/rubrae, resistance to local anesthetic drugs, acquired cutis laxa/premature skin aging, piezogenic papules of the heels, abnormalities of the uvula, absent/short lingual frenulum, and light blue sclerae. Subsequently, JHM was evaluated according to the BS [15]; in adults with a BS < 5 the presence of historical JHM was routinely investigated with the five-point questionnaire [31].

In addition, we selected a set of clinical features and signs related to almost all organ systems by adapting our clinical checklist routinely used for the assessment of patients with a suspicion of hEDS, in which the multisystemic involvement is already well recognized, even though not codified yet in the current nosology [1]. Further assessed musculoskeletal and orthopedic features included congenital hip dysplasia, sprains, (fixed) dislocations, subdislocations, articular pain, back pain, recurrent inflammatory soft-tissue lesions (i.e., bursitis, tendinitis, myofascial pain, carpal tunnel syndrome, epicondylitis, tenosynovitis, plantar fasciitis), walking difficulties, limited walking autonomy, temporomandibular joint dysfunction, high arched/narrow palate, marfanoid habitus (arm span to height ratio > 1.05), arachnodactyly (positive thumb/wrist sign), (non-surgical) pectus excavatum/carinatum, scoliosis, dorsal hyperkyphosis, cervical spine curvature anomalies, lumbar hyperlordosis/hypolordosis, minor asymmetry at lower limbs (anatomical or functional anisomelia) or other body areas, cubita/genua valga, halluces valgi, and pes planus/cavus. When available, the documented presence of disc hernias/protrusions, osteopenia/osteoporosis, and spondylolisthesis was recorded. In patients with osteopenia/osteoporosis the main primary causes (e.g. hormonal and vitamin D deficiencies, lifestyle, and medications) were investigated. Cardiovascular features that were systematically explored included varicose veins, Raynaud’s phenomenon, acrocyanosis, livedo reticularis, capillary fragility, and recurrent epistaxis or gingival bleeding. When possible, the presence of arterial aneurysms, progressive aortic root dilatation, aortic ectasia, valvular regurgitation with hemodynamic involvement, and mitral valve prolapse (MVP) was investigated by cerebral, thoracic, abdominal magnetic resonance imaging (MRI) and/or heart ultrasound. In patients with extensive easy bruising, routine coagulation tests were performed resulting normal in all. Assessed muscular features included hypotonia at birth, muscle hypotonia, recurrent myalgias and cramps, involuntary muscle contractions, and fibromyalgia. Investigated gastrointestinal features comprised gastroesophageal reflux, dysphagia, hiatal hernia, food intolerances, celiac disease, abdominal pain, defecatory dysfunction, delayed gastric/bowel/colonic transit, dolichocolon, and visceroptosis. Investigated uro-gynecological features included disabling dysmenorrhea, meno/metrorrhagia, post-partum hemorrhage, urinary stress incontinence, cervical insufficiency during pregnancy, and rectal or pelvic prolapse. Assessed neuropsychiatric features were delayed motor development, clumsiness, chronic fatigue, headache/migraine, impaired memory and concentration, paresthesia, cardiovascular dysautonomia, sleep disturbances, anxiety/panic/fears, allodynia, depression, and neuropathic pain. Immunological features included allergy/atopy, rhinitis/rhinoconjunctivitis, asthma, and atopic dermatitis.

Assessment between the presence/absence of investigated features and selected dichotomous variables, i.e., age at last examination < or ≥ 18 years and gender, was performed with the chi-square test with Yates’s correction or Fisher’s exact test whenever the count was insufficient. Analysis was carried out with the GraphPad Software and considering significant *p*-values when less than 0.05. In patients who have been evaluated at more than one visit, the 2017 diagnostic criteria were analyzed at the different ages, in order to appraise if they changed over time.

### Genetic testing

Molecular analysis was performed on genomic DNA purified from peripheral blood leukocytes by standard procedures. Most patients were characterized according to our previously reported molecular flow-chart based on (serial) single-gene Sanger sequencing [6]. In particular, all exons and intron-flanking regions of *COL5A1* (NM_000093.3, NP_000084.3), *COL5A2* (NM_000393.5, NP_000384.2), and exon 14 of *COL1A1* (NM_000088.4, NP_000079.2) were amplified by PCR with optimized primer sets (available upon request) followed by bidirectional Sanger sequencing with the BigDye Terminator v1.1 Cycle Sequencing kit on an ABI3130XL Genetic Analyzer (Life Technologies). Sequences were analyzed with the Sequencher 5.1 software (www.genecodes.com) and variants annotated by using the Alamut Visual software version 2.15 (Interactive Biosoftware, Sophia Genetics). Since 2018, massive parallel sequencing methods were applied in our laboratory and positive results confirmed by Sanger sequencing. For this study, patients were characterized by using an in-house NGS panel including all cEDS-associated genes. Briefly, two panel pools of the custom “connective tissue panel”, CTP (*COL5A1, COL5A2, COL1A1, COL1A2, COL3A1, B3GALT6, B4GALT7, SLC39A13, FKBP14, ADAMTS2, CHST14, DSE, FLNA, ACTA2, MYH11, MYLK, NOTCH1, MFAP5, PRKG1, SLC2A10, TGFBR1, TGFBR2, SMAD2, SMAD3, SMAD4, TGFB2, TGFB3, FBN1*) were generated with the AmpliSeq Designer tool (Thermo Fisher Scientific). Panel libraries were generated using the AmpliSeq Library kit 2.0, which includes reagents for generating amplicons with the Ion AmpliSeq primers and the Ion Xpress™ Barcode Adapters, following manufacturer’s protocols (Thermo Fisher Scientific). Library template preparation was performed with the Ion 520 & Ion 530 kit – OT2 on the Ion OneTouch 2 instrument starting from a pool of 15 barcoded libraries (8 μl of 100 pM pooled library) and sequenced on the Ion S5 instrument with the Ion 520 chip. Basecalling and sequence alignment against hg19 genome assembly were performed with the Ion Torrent Suite software v.5.0.2 and genetic variants were identified by the Ion Torrent Variant Caller v.5.0.2.1. Further analyses were performed with the Ion Reporter software 5.6. In patients without a pathogenic variant, deletion/duplication analysis of *COL5A1* was performed through Multiplex Ligation-dependent Probe Amplification (MLPA) analysis with the SALSA MLPA P331 and P332 Probe-Mixes, according to manufacturer’s instructions (MRC-Holland). All novel pathogenic variants were submitted to the EDS LOVD [27].

## Results

### General and molecular findings

**Fig. 3:**
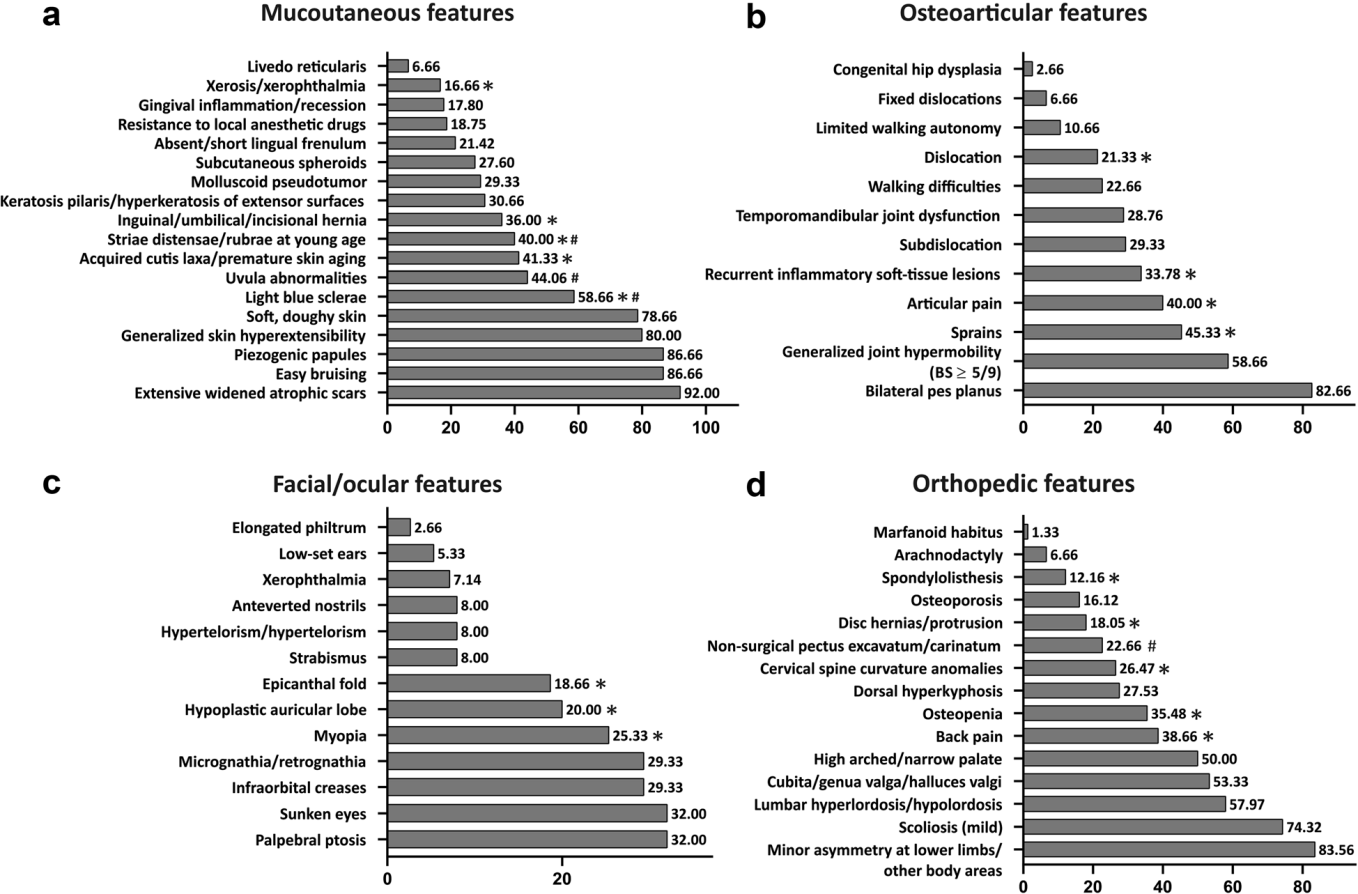
Frequencies of mucocutaneous (a), osteoarticular (b), facial/ocular (c), and orthopedic (d) features in the cEDS patients’ cohort. *presence of statistically significant differences by age; #presence of statistically significant differences by gender (for frequencies and *p*-values see Additional Table 2)

**Fig. 4:**
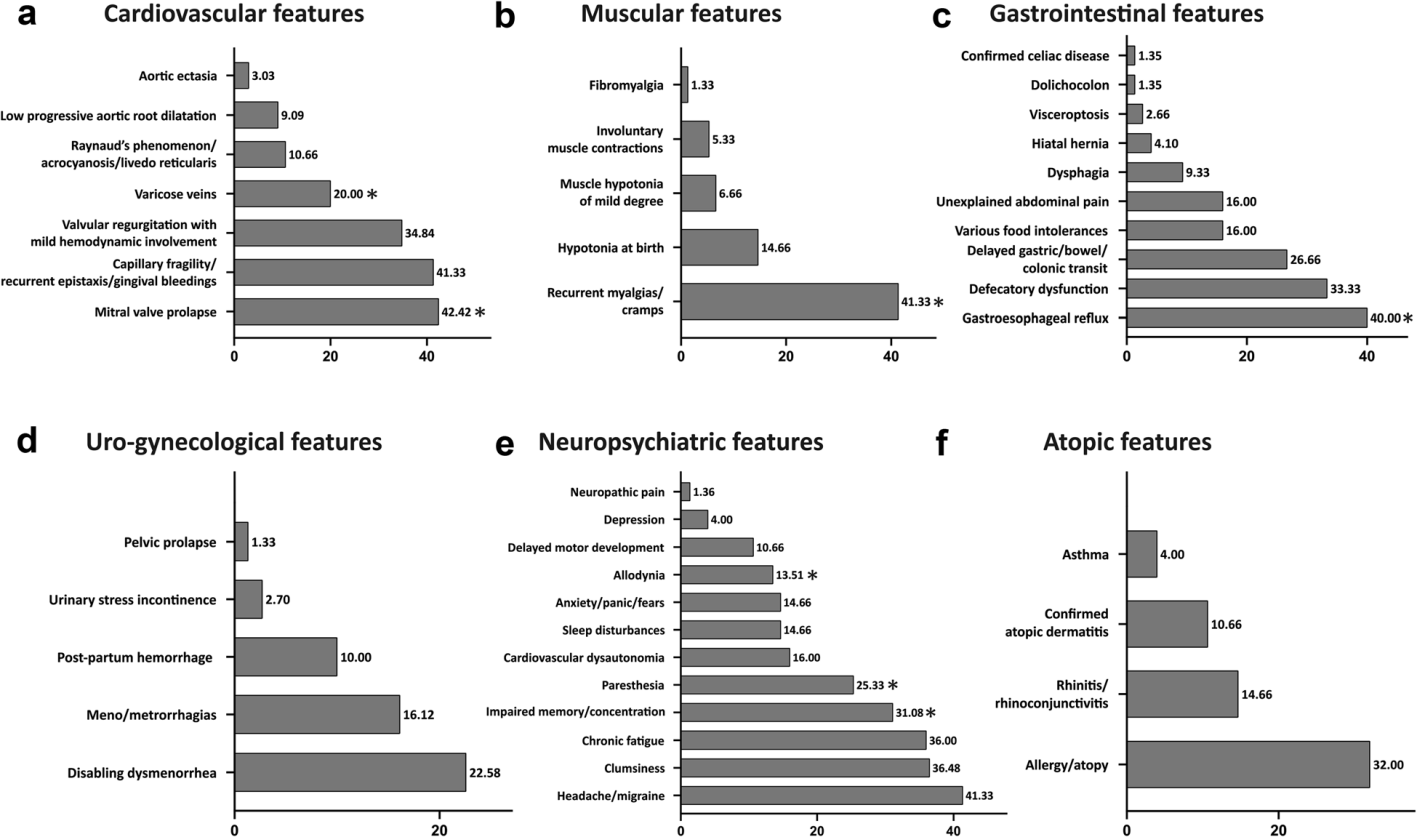
Frequencies of cardiovascular (a), muscular (b), gastrointestinal (c), uro-gynecological (d), neuropsychiatric (e), and atopic (f) features in the cEDS patients’ cohort. *presence of statistically significant differences by age; #presence of statistically significant differences by gender (for frequencies and p-values see Additional Table 2)

Among the 75 cEDS patients from 44 families described here, 45 were females (60%) and 30 were males (40%) (sex ratio: 1.5). By considering the index-cases, the sex ratio was 1.75 (28 females and 16 males). Forty-nine patients from 31 families were previously published in [6–8, 12], whereas 18 from 11 families were novel patients. Eight additional affected family members from 2 previously described pedigrees [6, 7] were also reported. Among the 49 earlier published patients that were included in the present study, 24 were reassessed at least once during scheduled follow-up visits (Additional Table 1). Age at last examination ranged from 3 to 67 years (mean 27; standard deviation [SD] 18.3). In particular, age range was 3 to 67 (mean 27.2; SD 17.8) for females, and 3 to 63 (mean 29.7; SD 19) for males. Patients younger than 18 years old were 31 (41.3%) and 44 (58.7%) patients were adults (≥18 years). In all new families, a *COL5A1* pathogenic variant was identified mainly by using the NGS CTP and submitted to the EDS LOVD. In particular, we disclosed a previously reported nonsense mutation [6] and 10 novel variants, i.e., 2 nonsense, 3 small deletions, and 1 multi-exon deletion, all predicted leading to haploinsufficiency, and 2 in-frame exon skipping splice variants, 1 glycine substitution, and 1 intermediate-sized duplication (63 bp) within the collagenous domain of the protein and with an estimated dominant negative effect. Detailed clinical and molecular features by single patient and the corresponding LOVD IDs are reported in Additional Table 1. Frequencies of selected clinical features are reported in Table 1, in Figs. 3 and 4, and in Additional Table 2.

**Table 1:** Major, minor, and minimal criteria according to the 2017 nosology of classical Ehlers-Danlos syndrome

	**Total**		**Males**		**Females**		***p*-value**^**A**^	**Patients < 18**		**Patients ≥ 18**		***p*-value**^**B**^
	N/T	%	N/T	%	N/T	%		N/T	%	N/T	%	
***Major Criteria***												
**Generalized skin hyperextensibility plus extensive atrophic scarring**	59/75	78.67	26/30	86.66	33/45	73.33	.274	24/31	77.41	35/44	79.54	.948
**Generalized joint hypermobility (BS ≥ 5)**	44/75	58.66	14/30	46.66	30/45	66.66	.138	19/31	61.29	25/44	56.81	.881
***Minor Criteria***												
**Easy bruising**	65/75	86.66	25/30	83.33	40/45	88.88	.728	29/31	93.54	36/44	81.81	.181
**Soft, doughy skin**	59/75	78.66	24/30	80.00	35/45	77.77	.954	24/31	77.41	35/44	79.54	.948
**Skin fragility (or traumatic splitting)**	67/75	89.33	28/30	93.33	39/45	86.66	.593	30/31	96.77	37/44	84.09	.129
**Molluscoid pseudotumors**	22/75	29.33	13/30	43.33	9/45	20.00	.055	10/31	32.25	12/44	27.27	.834
**Subcutaneous spheroids**	18/65	27.60	8/24	33.33	10/41	24.39	.623	10/29	34.48	8/36	22.22	.412
**Inguinal/umbilical/incisional hernia**	27/75	36.00	10/30	33.33	17/45	37.77	.883	18/31	58.06	9/44	20.45	**.002**
**Epicanthal folds**	14/75	18.66	6/30	20.00	8/45	17.77	.951	10/31	32.25	4/44	9.09	**.025**
**Complications of joint hypermobility (at least 1)**	70/75	93.33	28/30	93.33	42/45	93.33	.636	28/31	90.32	42/44	95.45	.683
Bilateral pes planus	62/75	82.66	25/30	83.33	37/45	82.22	.851	27/31	87.09	35/44	79.54	.588
Sprains	34/75	45.33	17/30	56.66	17/45	37.77	.169	8/31	25.80	26/44	59.09	**.008**
Pain	30/75	40.00	12/30	40.00	18/45	40.00	.809	3/31	9.67	27.44	61.36	**.001**
Subdislocations	22/75	29.33	8/30	26.66	14/45	31.11	.876	6/31	19.35	16/44	36.36	.181
Dislocations	16/75	21.33	9/30	30.00	7/45	15.55	.226	1/31	3.22	15/44	34.09	**.001**
Two or more of these complications	41/70	58.57	19/28	67.85	22/42	52.38	.298	10/28	35.71	31/42	73.80	**.003**
**Family history of a first degree relative who meets clinical criteria**	42/75	56.00	17/30	56.66	25/45	55.55	.886	10/31	32.25	32/44	72.72	**.001**
***Minimal Criteria***	59/75	78.66	26/30	86.66	33/45	73.33	.274	24/31	77.41	35/44	79.54	.948
Major criterion 1 plus major criterion 2	37/75	49.33	13/30	43.33	24/45	53.33	.540	14/24	58.33	23/44	52.27	.822
Major criterion 1 plus at least 3 minor criteria	22/75	29.33	13/30	43.33	9/45	20.00	.055	10/24	41.566	12/44	27.27	.963

*Abbreviations*: *N* number of patients presenting the investigated feature, *T* total number of patients in whom the feature was investigated

Significant *p*-values < 0.05 are in bold

A: *p*-values females vs males

B: p-values patients < 18 years vs patients ≥18 years

### Diagnostic criteria according to the 2017 EDS nosology and additional mucocutaneous, osteoarticular, and facial/ocular features

The two major criteria for cEDS, namely marked skin hyperextensibility plus extensive, widened atrophic scarring and gJHM, were present respectively in 78.7 and 58.7% of patients at last evaluation without any correlation with sex and age (Table 1).

Among minor criteria, skin fragility (89.3%), easy bruising (86.7%), and soft doughy skin (78.7%) were the most frequent cutaneous signs; molluscoid pseudotumors (29.3%) and spheroids (27.6%) were less common. These features did not show any significant difference between sex and age categories, whereas hernias and epicanthal folds (Fig. 2A) were more frequent in young patients (58.1% vs 20.5 and 32.3% vs 9.1%, respectively). At least one gJHM complication was present in 93.3% of patients, with bilateral pes planus (82.7%) as the most common feature without any correlation with sex and age. A higher prevalence in adults compared to younger individuals was clearly evident for the occurrence of sprains, especially of heels, fingers/wrist, and knees (59.1% vs 25.8%), joint and limb pain (61.4% vs 9.7%), and dislocations (34.1% vs 3.2%) that involved primarily shoulder, digits, hip, radius, and clavicles (Additional Table 1). Subdislocations were also more common in adults (36.4% vs 19.4%), although not statistically significant. Two or more of gJHM complications were present especially in adults (73.8% vs 35.7%). Fixed dislocations, particularly of shoulders, were sporadic events (6.7%). Likewise, chronic disabling pain was infrequent and patients, mainly adults, used principally nonsteroidal anti-inflammatory drugs or paracetamol on demand, whereas opioid therapy was very uncommon (Additional Table 2). Finally, the last minor criterion, namely family history of a first-degree relative meeting clinical criteria, was more common in adults (72.7% vs 32.3%).

Minimal criteria for a cEDS suspicion were present in 59/75 (78.7%) of patients; 49.3% showed both major criteria while 29.3% major criterion 1 together with at least three minor criteria; both combinations did not show sex- and age-related differences. Overall, 16/75 patients (21.3%) of our cohort did not fulfill the combined major criterion 1, i.e., 10 for the absence of generalized skin hyperextensibility, 1 for the lack of typical scarring, and 5 for the absence of both features (Additional Table 1). Localized skin hyperextensibility (less than 3 sites) was present in 9/75 patients and the complete absence of skin hyperextensibility in 6/75 (Additional Table 2). Concerning skin fragility, 92% of patients (69/75) showed extensive, widened atrophic scars and the most frequent types were papyraceous (65.3%), hemosiderotic (54.7%), and cigarette paper (40%) (Fig. 1A). The rate of hemosiderotic scars was significantly higher in young patients compared to adults (Additional Table 2). Further frequent (> 30%) mucocutaneous features were piezogenic papules of the heels (86.7%), light blue sclerae (58.7%), uvular abnormalities (44.1%), acquired cutis laxa/premature skin aging (41.3%), striae distensae/rubrae (40%), and keratosis pilaris/hyperkeratosis of extensor surfaces (30.7%). Among these, light blue sclerae were more common in young females, while acquired cutis laxa, premature skin aging, and striae distensae/rubrae were more recurring in adults, with the latter more frequent in females (Fig. 3a and Additional Table 2).

Further recognized osteoarticular features were recurrent inflammatory soft-tissue lesions (33.8%), more frequently observed in adults (48.8% vs 12.9%), and temporomandibular joint dysfunctions (28.8%). Walking difficulties (22.7%) and limited walking autonomy (10.7%) were rare and occurred more repeatedly in adults, though not statistically significant. Mobile patellae were present in 6/75 patients (8%). Congenital hip dysplasia was encountered only in 2 patients as well as spontaneous Achilles tendon rupture (Fig. 3b and Additional Tables 1 and 2).

Apart from epicanthal folds, additional midface/ocular signs were palpebral ptosis and sunken eyes (both 32%) and infraorbital creases (29.3%) (Fig. 2). Myopia (25.3%) was more common in adults. The other non-ocular facial features were mostly sporadic observations, except for micro-retrognathia (29.3%) and hypoplastic auricular lobe (20%), the latter with a predominance in young patients (Fig. 3c and Additional Table 2).

Considering that we reassessed 24 patients during follow-up (approximately 2–5 years after first examination), we had the possibility to evaluate if the major, minor, and minimal criteria changed over time in an individual single patient. Though the limited time period of observation (range 2–7 years), gJHM displayed an age influence. Indeed, in 14/24 patients (8 males and 6 females) a decrease of the BS was observed, and at last examination 5 of these patients (all males) displayed a BS < 5, hence not fulfilling the major criterion 2. The most striking difference was observed in a boy who showed at 10 years of age a BS of 9/9 that 4 years later changed to 2/9. Additional differences included a female that 7 years after the first assessment showed subcutaneous spheroids, and 2 patients (1 female and 1 male) who displayed after 4 years the presence of multiple molluscoid pseudotumors (Additional Table 1). While we did not observe any striking difference concerning skin hyperextensibility as well as the other minor criteria, in most patients, especially in adolescence, scars changed over time both in dimension and appearance. For instance, if no further injury occurred at the same area, large, thickened, raised, papyraceous, and hemosiderotic scars usually tended to resolve in smaller, flatter, and less hemosiderotic or even in cigarette paper scars (Fig. 1B).

### Orthopedic features

In almost all patients of our cohort we systematically evaluated 15 orthopedic features (Fig. 3d and Additional Table 2). The most recurrent (≥ 50%) were minor asymmetry at lower limbs or at other body areas (83.6%), mild scoliosis (74.3%), lumbar hyperlordosis/hypolordosis (57.9%), cubita/genua valga or halluces valgi (53.3%), and high arched/narrow palate (50%); all these features did not show any apparent sex and age bias. Osteopenia (T-scores between − 1.0 and − 2.4) was identified in 11 (8 females and 3 males) out 21 investigated adults (35.5%), whereas it was absent in 10 examined young individuals. The age range of patients with osteopenia was 29 to 58 (mean 41.5, SD 9.3). Osteoporosis (T-scores above − 2.5) was diagnosed only in 5/21 adults (16.1%, 4 females and 1 male, mean age 38.6, SD 11.4). Vitamin D deficiency was found in 4 patients and cholecalciferol treatment was commenced. Dorsal hyperkyphosis was present in 27.5% of patients without any sex- and age-related difference. A similar frequency was identified also for cervical spine curvature anomalies, but with a higher rate in adults compared to individuals aged below 18 years (36.5% vs 11.1%). Back pain (56.8% vs 12.9%), disk hernias/protrusions (27.9% vs 3.4%), and spondylolisthesis (2.5% vs 0%) were all more common in adults. Finally, non-surgical pectus excavatum/carinatum showed a higher prevalence in males compared to females (40% vs 11.1%) and in 1 of them marfanoid habitus and arachnodactyly were also evident. Other rare findings comprised short stature, dental malocclusion, radiological vertebral fractures, surgically treated congenital kyphoscoliosis, winged scapulae, ulnar deviation, brachydactyly, hypoplasia of the upper femur, arthrosis, osteoarthritis, club feet, pes cavus, and hindfoot deformities (Additional Table 1).

### Cardiovascular features

Apart from easy bruising, the most commonly observed cardiovascular sign in our cohort was MVP (42.4%), which was especially recognized in adults (56.4% vs 22.2), followed by capillary fragility/recurrent epistaxis/gingival bleeding (41.3%), valvular regurgitation with mild hemodynamic involvement (34.8%), varicose veins (20%), and Raynaud’s phenomenon/acrocyanosis/livedo reticularis (10.6%), all without any significant difference between sex and age categories, with the exception of varicose veins that were more frequent in adults. Non-progressive aortic root dilatation (9.1%) was detected only in adults, namely in 5/27 investigated males and in 1/39 females; as well as aortic ectasia (z score > 2) that was documented in 2 adult males, one patient carried the *COL1A1* c.934C > T p.(Arg312Cys) missense variant and the other a *COL5A1* null allele (Additional Tables 1 and 2, Fig. 4a). Spontaneous rupture of large arteries did not occur in our cohort. Additional sporadic findings included posterior cerebral artery hypoplasia, intracranial aneurysm, vertebral artery tortuosity, atrioventricular block, tricuspid valve prolapse, interatrial septal aneurysm, bicuspid aortic valve, delayed right ventricular conduction, left ventricular thickening, ventricular arrhythmia, tachycardia with extrasystoles, arteriovenous fistula, and deep venous thrombosis (Additional Table 1).

### Muscular features

Among the 5 investigated muscular signs (Additional Table 1 and Fig. 4b), the most common issues were recurrent myalgias and cramps (41.3%) that affected mainly adults (52.3% vs 25.8%). Hypotonia at birth was infrequent (14.7%) as well as muscle hypotonia of mild degree (6.7%) and involuntary muscle contractions (5.3%). Confirmed fibromyalgia was present only in 1 patient with the most severe form of cEDS, carrying a structural mutation in *COL5A2* (Patient 14 in Additional Table 1), who also suffered from recurrent muscle hematomas and ruptures.

### Gastrointestinal features

In almost all individuals of our cohort we assessed 11 gastrointestinal features (Additional Table 2 and Fig. 4c). The most recurrent problem, especially in adults, was gastroesophageal reflux (52.3% vs 22.6%). Treatment with proton pump inhibitors was resolutive in most cases, only 3 patients required fundoplication (Additional Table 1). Defecatory dysfunction (33.3%), delayed gastric/bowel/colonic transit (26.3%), various food intolerances, unexplained abdominal pain (both 16%), and dysphagia were less common and did not show any sex and age bias. Sporadic findings comprised hiatal hernia, visceroptosis (gastroptosis, enteroptosis), dolichocolon, confirmed celiac disease, and gastric ulcer (Additional Table 2). Inflammatory bowel disease was not recognized. In one patient a postnatal intestinal perforation with meconium peritonitis requiring ileostomy occurred.

### Uro-gynecological features

Among females in reproductive age (26 adults and 5 aged below 18 years), disabling dysmenorrhea (22.6%) and meno/metrorrhagias (16.1%) were the most frequent gynecological issues (Additional Table 2 and Fig. 4d). One patient suffered from amenorrhea since the age of 24 (Patient 11 in Additional Table 1). Twenty out of 31 women had at least one pregnancy and 8 of them had multiple pregnancies. Fourteen of the children were affected. Multiple miscarriages occurred in 4 patients. Post-partum uterine hemorrhage and a third-degree vaginal laceration during delivery occurred in 2 and 1 patients, respectively. Premature rupture of membranes with preterm delivery was present in 1 patient with an affected fetus; 16 women had uncomplicated pregnancies and delivery (Additional Table 1).

In the entire patients’ cohort, urinary stress incontinence was very rare (2.7%). In the most severely affected patient (Patient 14), both rectal and urethral prolapse together with recurrent hemorrhagic cystitis occurred. Further occasional issues were microhematuria, urinary retention, testicular cysts, bilateral mobile testis, pelvic varicocele, cryptorchidism, and bladder diverticula and hypoplasia (Additional Table 1).

### Neuropsychiatric features

In order to explore the neurological, psychological and emotional dysfunctions in cEDS, we investigated 12 features (Additional Table 2 and Fig. 4e). The most common problematic was headache/migraine, which was reported by 41.3% of patients without significant sex- and age-related differences, even if it affected more frequently adult females. Motor clumsiness was reported by 36.5% of patients as well as chronic fatigue (36%), the latter was more frequent in adults (45.5% vs 22.6%), although not statistically significant. Abnormal sensation of the skin (e.g. formication, tingling, pricking, chilling, burning, and numbness) was described by 25.3% of patients, especially by adults (36.4% vs 9.7%). Paresthesia was most commonly occurring in arms, legs, and abdomen and was usually painless and transient, except for 5 females who mentioned chronic formication (Additional Table 1). A documented cardiovascular dysautonomia was present in 16% of patients, particularly in adult females, although not reaching a statistic significance. The most recurrent symptom was orthostatic intolerance and in 9 patients tilt-table testing resulted positive. Further associated symptoms were mood swings, fatigue, problems with concentration and memory, gastrointestinal complications, anxiety/panic/fears, and sleep disturbance (Additional Table 1). These latter issues were both reported also by patients without cardiovascular dysautonomia with an overall frequency of 14.7% without any sex and age bias. Allodynia (13.5%) was more recurrent in adults (20.5% vs 3.3%). The occurrence of either mechanical (especially static), thermal (hot and cold), or movement (joints and/or muscles) allodynia was referred. Delayed motor development, usually associated with primary hypotonia (Additional Table 1), was present in 10.7% of patients without any significant variance between sex and age categories. Chronic depression requiring pharmacological treatment was diagnosed in 3 patients (1 adult female and 2 adolescents), one of the latter showed a self-injurious behavior and attempted suicide (Additional Table 1). Chronic neuropathic pain was present only in the most severely affected patient (Patient 14). Further sporadic features were epileptic seizures (2 patients from the same family), mild hearing loss, absence of sphincter control, and developmental coordination disorder affecting fine and gross motor skills (Additional Table 1).

### Atopic features

In the entire cohort, immunological issues were quite rare (Additional Table 2 and Fig. 4F). Allergy/atopy (32%) was the most recurrent problem followed by recurrent rhinitis/rhinoconjunctivitis (14.7%) and confirmed atopic dermatitis (10.7%). Asthma afflicted only 3 out of 75 patients. The most common allergens were various foods, pollen, animal dander (especially cat hair), and dust mites. Treatment with corticosteroids was resolutive in almost all patients and anaphylaxis did never occur. One patient suffered from chronic sinusitis (Additional Table 1).

## Discussion

This study on a cohort of 75 molecularly proven cEDS patients aimed to validate the revised diagnostic criteria and explore the multisystemic involvement of the disorder by comparing types and incidences of the numerous clinical manifestations at different ages and with those observed in other EDS subtypes, in order to delineate natural history and assist differential diagnosis. Our results confirm the notion that cEDS is basically characterized by cutaneous and articular involvement, even though none of their hallmarks is represented in 100% of cases, and show that osteoarticular and musculoskeletal complaints as well as gastrointestinal, uro-gynecological, neuropsychiatric, and cardiovascular associated symptoms (and disorders) are not so prominent compared to other EDS subtypes.

Albeit the cEDS-specific triad, i.e., widened, atrophic scars (92% of patients and ~ 95% of probands), marked skin hyperextensibility (80% of patients and ~ 82% of probands), and gJHM (BS ≥5, ~ 59% of patients and ~ 66% of probands), is highly predictive for molecular confirmation of the diagnosis, this combination was observed only in ~ 49% of patients and in ~ 55% of probands. Hence, the suspect of cEDS is not always driven by the traditional criteria but is rather gestaltic and based on the overall clinical presentation. Our findings offer future perspectives for a revision of the EDS nosology in terms of diagnostic criteria. The observation that in our cohort only ~ 79% of patients and ~ 82% of probands fulfilled the currently defined minimal criteria for a suspicion of the disease, which require the presence of the combined major criterion 1, indicates that their strict application could lead to a lack of diagnoses. Hence, we recommend that atrophic scarring should be considered independently from skin hyperextensibility as an alone-standing major criterion 1. We also propose that minimal criteria prompting genetic testing should be typical cEDS scars (at more than 2 sites) plus either generalized skin hyperextensibility (at least 3 of the currently defined areas and according to their cut-off values) and/or gJHM (BS ≥5), and/or 3 of the currently defined minor criteria, to which orthopedic issues may be added. In view of intrafamilial variability, a family history with a documented pathogenic variant is sufficient for genetic testing in individuals not fulfilling the criteria.

These suggestions are supported first by the fact that in our sample abnormal scarring was undoubtedly the most common sign. Although scarring was variable in clinical appearance and affected sites and is shared by other EDS subtypes and HCTDs [1, 9, 28–30, 32–35], scars should basically be considered representative of cEDS. Indeed, in our cohort, scars were typically numerous, large, papyraceous, and hemosiderotic. The findings that the rate of hemosiderotic scars was significantly higher in young patients and that adults often presented small and flat scars suggest an evolving phenotype and natural history of scarring that usually becomes less severe in adulthood (Fig. 1) and is likely influenced by patients’ habits. Defective scarring in cEDS is always secondary to soft tissue traumas, which occur more likely in childhood in exposed areas such as forehead, knees, and elbows, and in those near the underlying bones (e.g. pretibial region), due to skin fragility joined with a defective wound healing. The reevaluation of scars in single individuals over time (Fig. 1) corroborates the concept of an evolving phenotype and suggests that once a cEDS diagnosis is made (or in the presence of a positive family history) and management guidelines for prevention of primary manifestations are provided (e.g. avoiding contact sports or wearing protective pads or bandages over forehead, knees, and shins during activities), scarring generally becomes less significant, except for severe cases.

The proposal to modify the current diagnostic criteria also derives from the observation that in 15 out of 16 patients of our cohort who did not satisfy the major criterion 1 (and consequently neither the minimal criteria for genetic testing), this was due to the absence of generalized skin hyperextensibility. Ten of these patients showed the typical cEDS scars and 5 of them also had an affected family member fulfilling the criteria, thus prompting molecular analysis. Likewise, among the 5 patients who did not show both features of criterion 1, 3 had a first-degree parent meeting the criteria and one an affected mother with positive skin hyperextensibility, although without the typical cEDS scars [8]. The most uncertain patient was a sporadic female (Patient 23 in Additional Table 1) who presented only a single, small atrophic scar and localized skin hyperextensibility at elbows and knees. In addition to this latter sign, the presence of gJHM and its complications and further minor signs prompted molecular analysis that revealed a pathogenic *COL5A1* variant. This case highpoints that while molecular diagnosis in patients with a full-blown phenotype is mainly confirmatory, in those with an incomplete presentation it turns out to be fundamental, since these individuals might not be diagnosed or even be misdiagnosed. Indeed, considering the clinical overlap not only between the different EDS subtypes but also with other HCTDs [1, 9, 28, 30, 32, 35–49], differential diagnosis is not always forthright. Differential diagnosis includes the molecularly unsolved hEDS that shares with cEDS gJHM and more than a few (muco) cutaneous signs; however, hEDS patients usually show a lower degree of scarring and skin hyperextensibility and much more striking gJHM complications [1, 7, 28, 29, 50]. Molluscoid pseudotumors and subcutaneous spheroids are highly diagnostic of cEDS, even if they were rarely observed in our patients’ cohort. Signs of premature skin aging, including acquired cutis laxa of the extremities and skin wrinkling, although less specific, might also help in the differential with hEDS, especially in adults. The additional mucocutaneous features, including abnormal skin texture, easy bruising, hernias, piezogenic papules, light blue sclerae, uvular abnormalities, striae distensae/rubrae, keratosis pilaris/hyperkeratosis of extensor surfaces, absent/short lingual frenulum, resistance to local anesthetic drugs, and gingival inflammation/recession, are almost all shared not only with hEDS but also with other EDS subtypes and HCTDs [28, 29]. As such, the recording of these features at physical examination might reinforce the suspect of systemic disorder, but their low specificity does not attend for confirmatory genetic testing. In cases compatible with an autosomal recessive transmission, differential diagnosis includes the rare classical-like EDS (clEDS) type 1, a.k.a. *TNXB* deficiency [51], and the recently defined clEDS type 2 caused by biallelic variants in *AEBP1* [52]. The clEDS type 1 is generally distinguishable from cEDS for the absence of atrophic scarring [28, 45, 53–57], whereas a more severe multisystemic presentation in clEDS type 2 should assist the differential diagnosis with cEDS [43, 52, 58–60]. The dermatosparaxis (*ADAMTS2*) [61], cardiac-valvular (*COL1A2*) [62–64], kyphoscoliotic (*PLOD1*, *FKBP14*) [65, 66], and arthrochalasia (*COL1A1*, *COL1A2*) [67, 68] EDS subtypes, also sharing with cEDS several cutaneous and articular issues, are mostly distinguishable for the presence of specific hallmarks [1, 9, 16].

Concerning the other major criterion of cEDS, gJHM remains undeniably characteristic of the disorder, even if in our sample a BS ≥5 was identified in less than 60% of patients and it is common to all EDS subtypes and other HCTDs [1, 9, 28, 30, 36–39, 47, 50, 69, 70]. In our previously published smaller cEDS cohort [7], a lower incidence of gJHM was observed in adults compared to younger individuals, suggesting that age affects JHM also in cEDS, as more widely demonstrated in hEDS and in which an age-dependent BS was introduced in the revised diagnostic criteria [1, 50, 69, 71–73]. In the present larger cEDS cohort, no statistically significant difference emerged between age at ascertainment and BS ≥5 (~ 57% in adults vs ~ 61% in young individuals), suggesting a different natural history of JHM compared to hEDS. Indeed, although patients’ reassessment at different ages showed a decrease of the BS in 12/24 investigated patients, only in 5 males it resulted below the cut-off value of 5/9. A different age influence and disease course in cEDS seems to occur also for joint instability complications, such as dislocations, sprains, and soft-tissue injuries. In hEDS, it was previously shown that there were no overt associations between age and their frequency and location, except for soft-tissue injuries that exhibited a higher rate in adults [73]. In our cEDS patients’ cohort, these gJHM complications as well as articular pain, which in general was of mild degree, were instead all more frequently observed in adulthood. However, gJHM complications showed an overall lower rate with the exclusion of pes planus (present in most patients) and recurrent inflammatory soft-tissue injuries, among which plantar fasciitis, bursitis, and tenosynovitis were particularly frequent. Furthermore, dislocations usually resolved spontaneously and were in general easily managed by the affected individual. Hence, in cEDS, a BS ≥ 5 and mild to moderate joint instability complications seem valid markers in adults, especially in those with a minor or naturally diminished cutaneous phenotype (Fig. 1). In contrast, gJHM and its related issues might lack both sensitivity and specificity in children, in whom, however, the typical cutaneous hallmark is nearly always plainly manifest. Furthermore, the presence of epicanthal folds and/or infraorbital creases or an association of the other eye findings and/or non-ocular features plus abnormal facial scars seems to denote a further distinctive gestaltic presentation of young cEDS patients, which usually attenuates or even vanishes over the years (Fig. 2). Musculoskeletal involvement was an interesting finding in our cohort, due to the high prevalence of some related issues, thus suggesting that the registration of these features might strengthen the suspect of cEDS, although with a low specificity. This is particularly true for orthopedic features, considering that in our cohort minor asymmetry at lower limbs and/or other body areas, scoliotic attitude/mild scoliosis, lumbar hyperlordosis/hypolordosis, valgus deformities of elbows, knees, and feet, and high arched/narrow palate were all observed with a frequency above 50% without any sex and age bias. Hence, it is reasonable to propose that the presence of 3 or more of these issues might be considered as an additional minor criterion in a future nosology revision. Osteopenia and osteoporosis, dorsal hyperkyphosis, cervical spine curvature anomalies, pectus deformities, disk hernias/protrusions, and spondylolisthesis as well as severe back pain (≥ 7 NRS numeric pain rating scale), which in general associated with the presence of radiological vertebral fractures (Additional Table 1), were less frequent but all more recurrent in adults, except for dorsal hyperkyphosis and pectus anomalies. Osteoarthritis, which has been postulated as a long-term consequence of JHM and related altered joint biomechanics [69], was instead only a sporadic finding in our cEDS cohort as well as marfanoid habitus. Our results partly support the still controversial impression that bone quality might be impaired in cEDS [74–78], although undoubtfully less significant compared to other EDS subtypes (e.g. spondylodysplastic, arthrochalasia, kyphoscoliotic, and classical-like type 2 EDS) [9, 43, 44, 48, 49, 52, 59, 65, 68] or skeletal dysplasia [39], and suggests a potential involvement of skeletal fragility in determining a poorer quality of life (QoL) in adult patients. Our findings emphasize a milder phenotype and disease course in cEDS compared to hEDS [50, 69] and other EDS subtypes (e.g. clEDS type 1 and 2, kyphoscoliotic, spondylodysplastic, myopathic, and musculocontractural EDS) [9] also regarding muscular and neuropsychiatric involvement. Indeed, in our cohort, apart from myalgias and cramps that affected about 50% of adults, primary (causing delayed motor development and clumsiness in few cases) or acquired muscle hypotonia/weakness (always of mild degree), and involuntary muscle contractions were quite rare; fibromyalgia was only a sporadic observation. These muscular complications, except primary hypotonia, are instead very frequent in hEDS and they intensely contribute to the poor QoL in adulthood [50, 69], when patients usually show generalization and progressive chronicity of musculoskeletal pain, which is often diagnosed as fibromyalgia [79], summation of other forms of chronic pain, such as pelvic pain (in women) and migraine as well as exacerbation of fatigue, together with additional complaints including paresthesia, mixed functional gastrointestinal disorders, and orthostatic intolerance [71]. In our cEDS cohort, headache/migraine and chronic fatigue were referred respectively by ~ 41% and ~ 36% of cEDS patients, especially by adults even though not statistically significant, whereas impaired memory and concentration and paresthesia were both more recurrent in adults (~ 40% vs ~ 10%). Cardiovascular dysautonomia as well as allodynia and sleep disturbance were less frequent (~ 15%); 5 adults mentioned chronic pain needing opioid therapy, whereas neuropathic pain was present only in the most severely affected patient. While fatigue affects about 1/3 of the general population [80], the chronic fatigue syndrome, defined as fatigue lasting longer than 6 months, occurs only in ~ 1% of the general population [81, 82] and is usually associated with impaired memory, cognitive deficits, muscle and joint pain, headaches, non-restorative sleep, post-exertional illness as well as psychological issues [83–85]. The discreetly high incidence of chronic fatigue observed in our cEDS cohort is in line with previous observations that emphasized that in EDS this issue is more common than in the general population and likely contributes to the patients’ functional impairment, psychological distress, and decreased QoL [86–89]. Like in the general population, in cEDS fatigue is multifactorial with contributing factors including articular pain, headache, sleep disturbance, and abnormally low blood pressure often associated with postural orthostatic tachycardia syndrome (POTS) or neurally mediated hypotension (NMH), nutritional deficiency, medications, and/or allergies (present in 32% of our patients) [80, 81, 87, 90–95]. Cerebrospinal fluid leak, which has been reported in cEDS as a cause of postural hypotension and headache [96], was not diagnosed in our cohort (e.g. increase in headache following Valsalva maneuver or reduction of headache when the patient takes a prone position) and only few patients with persistent migraine were investigated by imaging techniques with negative results. The first evidence for a possible link between EDS and autonomic dysfunction was published by Rowe et al. [86], who studied eleven pediatric patients (cEDS and hEDS) all showing either POTS or NMH. The concept was later reinforced by further studies that demonstrated a significant increase of the rate of systemic dysautonomic symptoms in hEDS [31, 97]. In our cEDS cohort, cardiovascular dysautonomia, although not particularly frequent, can explain orthostatic intolerance, palpitations, and tachycardia as well as some neurological secondary manifestations including fatigue, mood swings, and memory and concentration troubles. Besides, it has been proposed that in EDS dysautonomia might contribute also to features affecting the gastrointestinal and urinary systems (e.g. gut dysmotility and underactive/overactive bladder) as well as to certain psychological traits [98, 99]. Indeed, psychological dysfunction and emotional problems (e.g. depression, anxiety, panic, fears, affective disorder, low self-confidence, negative thinking, and hopelessness) are common features among EDS patients [92, 100–105]. Some of these issues, which may worsen the pain experience as well as other organ system manifestations (gastrointestinal and autonomic), were encountered also in our cohort with anxiety, panic, and/or fears that were referred by ~ 15% of patients. Chronic depression requiring pharmacological treatment was instead rare.

Concerning gastrointestinal involvement, its relevance in EDS and other HCTDs is well recognized [31, 69], whereas, until now, these issues were only slightly investigated in cEDS [106]. A previous study, which intended to explore the relationship between hEDS or gJHM with gastrointestinal disorders, showed that treatment-resistant functional issues such as gastroesophageal reflux, dysphagia, heartburn, swelling, constipation, recurrent abdominal pain, and irritable bowel syndrome, might be observed in 1/3 to 3/4 of patients with an increasing rate by age [107]. In our cEDS cohort, the most common feature was gastroesophageal reflux, especially in adults (~ 52% vs ~ 22%); however, unlike hEDS, it was mostly responsive to treatment with proton pump inhibitors and/or nizatidine. Constipation with or without other features of voiding dysfunction was often the initial sign of gastrointestinal involvement, but problems such as hemorrhoids, rectal bleeding, and anal fissures were uncommon and more serious complications such as rectal prolapse or fecal impaction did not occur. Likewise, hiatal hernia, visceroptosis, dolichocolon, and celiac disease, for which increased rates were described in hEDS [108, 109], were only sporadic observations in our sample.

In females with EDS, gynecologic complaints, such as mucosal problems with their genital area [69] , heavy menstrual or intermenstrual bleedings [110] and painful intercourse [71, 111] are commonly encountered with an incidence ranging from 30 to 75%. The frequencies of disabling dysmenorrhea (~ 23%) and meno/metrorrhagias (~ 16%) observed in our cohort are basically in line with these previous observations, but the high incidence of these issues also in the general population does not allow to draw relevant conclusions. Pelvic floor disorders including urinary stress incontinence, pelvic organ prolapse, and other sensory and emptying abnormalities were very uncommon among our affected females. As in the general population, childbirth has a very substantial impact on a woman’s probability of these complaints. Several case-control studies in the past suggested that EDS is associated with pelvic floor disorders [111–113], but most of these studies have not controlled for childbirth history or age and included patients affected by various types of EDS. Furthermore, several additional pregnancy-related complications have been more commonly reported in women with EDS in some studies [114] but as often, not validated in others [110, 115, 116]. Particularly for cEDS, it was suggested that pregnancy places both the newborn and the mother at risk for complications, which are assumed to be more frequent than in the normal population [16]. However, no comprehensive studies exist, and it is therefore very difficult to quantitate the incidence and related risk of each complication in affected individuals. In our cohort, most women had uncomplicated pregnancies (even multiple), deliveries, and postpartum period. Indeed, miscarriages, premature rupture of membranes, preterm birth, vaginal laceration during delivery, and post-partum uterine hemorrhage were rare or sporadic as well as rectal and urethral prolapse that occurred only in the most severely affected individual. Uterine rupture in the last trimester of pregnancy, which is particularly significant in vEDS as it associates with a high mortality rate (~ 5%) in affected females [46, 117, 118], did never happen in our patients. Overall, though the small sample size, pregnancy-associated complications seem to be unusual in cEDS. Accurate monitoring of women throughout gestation and in the postpartum period is still recommended, also considering that pregnancy was one of the most important sources of anxiety in our cEDS females.

The vascular aspect of EDS in general remains the major fear for the patients and their clinicians, especially regarding the possibility of arterial aneurysms, dissections and ruptures of medium-sized and large arteries. These life-threatening complications are principally the hallmark of vEDS together with gastrointestinal and uterine fragility, but they may occur also in other EDS subtypes, including rare individuals with a severe form of cEDS [16]. However, an exact estimation of prevalence and risk of these vascular complaints and possible genotype-phenotype correlations in cEDS are still missing [16, 119]. In particular, either the specific c.934C > T, p.(Arg312Cys) variant in *COL1A1* or *COL5A1* variants causing glycine substitutions at the C-terminal end of the triple helix domain have been associated with propensity to severe arterial events in early adulthood, but these assumptions are still a matter of debate due to the limited number of reports [10–14, 22, 25]. In our cEDS cohort, easy bruising, which is present to a variable degree in all EDS subtypes, was the most common finding (~ 87%). A bleeding tendency, despite normal coagulation status, manifesting either as meno/metrorrhagia and (less frequently) postnatal or peri-operative hemorrhage or as gingival bleeding/recurrent epistaxis (~ 41%) were also common. Varicose veins (~ 20%) and Raynaud’s phenomenon/acrocyanosis/livedo reticularis (~ 11%) were relatively unusual. All these features, except varicose veins, did not display any sex and age bias. MVP showed instead a significantly higher incidence in adults (~ 56% vs ~ 22%). Nevertheless, MVP was utmost without or of little clinical consequence, valvular regurgitation with mild hemodynamic involvement was rare (~ 35%), and none of our patients required surgical intervention, in line with earlier reports [16, 20]. Similarly, non-progressive aortic root dilatation was infrequent (~ 9%) as well as aortic ectasia (only 2 adult males) and tricuspid valve prolapse and bicuspid aortic valves were sporadic findings, thus suggesting that regular routine echocardiograms to assess for valvular diseases and aortic root dilatation may not be strictly necessary unless warranted by presence of symptoms or family history. MVP was formerly considered a common feature of all EDS subtypes and many other HCTDs, but that happened prior to the establishment of the more rigorous criteria for the diagnosis of MVP. Since then, some studies showed no significant increase in the incidence of MVP in EDS compared to the general population [18–20], whereas others disclosed a higher MVP frequency (28–67%) [120, 121, 122]. Considering the rate of MVP observed in our cEDS cohort and the knowledge that the mitral valve depends upon collagen for its tensile strength and that myxomatous MVP is characterized by disruption of the collagen layer with expansion of glycosaminoglycans within the middle layer of the valve [123], it is reasonable to consider MVP as a potential clue for cEDS, although the true clinical significance is so far unknown. The most noteworthy vascular complaints in our cohort were two aortic ectasias, an arteriovenous fistula, an interatrial septal aneurysm and an intracranial aneurysm; all these issues, except one of the two z scores > 2, were recognized in adults harboring a *COL5A1* null allele. Spontaneous rupture of large arteries was not observed at all and, in particular, neither in the 6 patients with the *COL1A1* p.(Arg312Cys) missense variant nor in 2 individuals with different *COL5A1* glycine substitutions at the C-terminal domain (Additional Table 1), which represent variants previously associated with a possible higher risk of vascular events [16]. The fact that i) these 8 patients were diagnosed years ago [12], ii) none of them originally experienced major vascular events (1 individual with the *COL1A1* variant showed aortic ectasia, uncomplicated tortuous vertebral arteries, and left ventricular thickening), and iii) all underwent periodical vascular surveillance that resulted negative at the time of writing, suggests that the supposed genotype-phenotype correlations of these variants should not be considered valid in all cases. The finding that major vascular complaints and/or rupture can be rarely recognized also in patients with different *COL5A1* variants [5, 21, 23, 24, 26] advises that a cerebral, thoracic, and abdominal MRI at diagnosis might be considered in a precautionary way for all adult cEDS patients. Once significant changes in arterial caliber are not detected and in pediatric patients, a doppler-ultrasound of sopra-aortic branches and abdominal arteries, and a heart doppler-ultrasound with aortic root, arch, and ascending aorta evaluation every 4–5 years might be enough together with cardiovascular risk factors monitoring. Overall, despite the few above-mentioned reports and the suggestion for a possible association between type V collagen defects and cardiovascular fragility demonstrated in *Col5a1* and *Col5a2* knock-out mice [124, 125], our results suggest that the risk for severe vascular complications seems to be fairly low in humans harboring a type V collagen defect and the presence of additional, yet unknown, genetic modifiers in families with a severe vascular phenotype is reasonable.

## Conclusions

In conclusion, we explored the accuracy of the 2017 nosology for the cEDS diagnosis in a cohort of patients from different ages and sexes with a defined molecular defect and tried to offer a full picture of the multisystemic involvement and some views on the natural history of this condition. Our findings define the value of atrophic scars, skin hyperextensibility, JHM and its complications in selecting patients for molecular testing and the relevance of the additional clinical signs associated with cEDS, which should contribute to the rapid ascertainment of cEDS patients and facilitate differential diagnosis. Promptly recognizing among the different EDS subtypes and confirmatory genetic testing is indeed increasingly important as these disorders are characterized by different natural histories and prognoses. Moreover, our results suggest the need of a future update of the currently defined diagnostic criteria for cEDS. Additional studies on large cEDS cohorts are expected to confirm the limited multisystemic involvement and rather favorable natural history of cEDS and to explore possible genotype-phenotype correlations, which would permit to establish management guidelines.

## Supplementary information

**Additional file 1: Table 1.** Clinical and molecular features of all cEDS patients.

**Additional file 2: Table 2.** Multisystemic features in the cEDS patients’ cohort by sex and age.

## Data Availability

All data generated or analyzed during this study are included in this published article and its Additional files.

## Abbreviations

BS: Beighton score
cEDS: Classical Ehlers-Danlos syndrome
clEDS: Classical-like Ehlers-Danlos syndrome
CTP: Connective tissue panel
EDS: Ehlers-Danlos syndrome
gJHM: Generalized joint hypermobility
HCTD: Heritable connective tissue disorder
hEDS: Hypermobile Ehlers-Danlos syndrome
JHM: Joint hypermobility
LOVD: Leiden Open Variation Database
MLPA: Multiplex ligation-dependent probe amplification
MRI: Magnetic resonance imaging
MVP: Mitral valve prolapse
NGS: Next generation sequencing
NMH: Neurally mediated hypotension
POTS: Postural orthostatic tachycardia syndrome
QoL: Quality of life
SD: Standard deviation
vEDS: Vascular Ehlers-Danlos syndrome
