# Supplementary material for: Multisystemic manifestations in a cohort of 75 classical Ehlers-Danlos syndrome patients: natural history and nosological perspectives
Source: Orphanet J Rare Dis. 2020 Jul 31;15:197. doi: 10.1186/s13023-020-01470-0 (PMC7393722; doi:10.1186/s13023-020-01470-0)
Supplement: Supplementary file 2 — Additional file 2: Table 2. Multisystemic features in the cEDS patients’ cohort by sex and age. [file 13023_2020_1470_MOESM2_ESM.docx]

| **Additional Table 2. Multisystemic features in the cEDS patients’ cohort by sex and age** | | | | | | | | | | | | | | | | | | | | | | | | |
| --- | --- | --- | --- | --- | --- | --- | --- | --- | --- | --- | --- | --- | --- | --- | --- | --- | --- | --- | --- | --- | --- | --- | --- | --- |
|  | **Total** | | | | | **Males** | | | **Females** | | | | | **p-value^A^** | **Patients <18** | | | **Patients ≥18** | | | | | | **p-value^B^** |
|  | **N/T** | **%** | | | | **N/T** | **%** | | **N/T** | | | | **%** |  | **N/T** | | **%** | **N/T** | **%** | | | | |  |
| **Mucocutaneous features** | | | | | | | | | | | | | | | | | | | | | | | | |
| Generalized hyperextensible skin (at least 3 sites) | 60/75 | 80.00 | | | | 26/30 | 86.66 | | 34/45 | | | | 75.55 | .376 | 24/31 | | 77.41 | 36/44 | 81.81 | | | | | .860 |
| Localized hyperextensible skin (less than 3 sites) | 9/75 | 12.00 | | | | 2/30 | 6.66 | | 7/45 | | | | 15.55 | .425 | 4/31 | | 12.90 | 5/44 | 11.36 | | | | | .873 |
| Absent skin hyperextensibility | 6/75 | 8.00 | | | | 2/30 | 6.66 | | 4/45 | | | | 8.88 | .930 | 3/31 | | 9.67 | 3/44 | 6.81 | | | | | .986 |
| Extensive widened atrophic scars | 69/75 | 92.00 | | | | 29/30 | 96.66 | | 40/45 | | | | 88.88 | .434 | 30/31 | | 96.77 | 39/44 | 88.63 | | | | | .397 |
| Small atrophic scar(s) | 6/75 | 8.00 | | | | 1/30 | 3.33 | | 5/45 | | | | 11.11 | .434 | 1/31 | | 3.22 | 5/44 | 11.36 | | | | | .397 |
| Papyraceous scars | 49/75 | 65.33 | | | | 23/30 | 76.66 | | 26/45 | | | | 57.77 | .150 | 19/31 | | 61.29 | 30/44 | 68.81 | | | | | .710 |
| Hemosiderotic scars | 41/75 | 54.66 | | | | 20/30 | 66.66 | | 21/45 | | | | 46.66 | .142 | 23/31 | | 74.19 | 18/44 | 40.90 | | | | | **.008** |
| Cigarette paper scars | 30/75 | 40.00 | | | | 13/30 | 43.33 | | 17/45 | | | | 37.77 | .809 | 9/31 | | 29.03 | 21/44 | 47.72 | | | | | .165 |
| Easy bruising | 65/75 | 86.66 | | | | 25/30 | 83.33 | | 40/45 | | | | 88.88 | .728 | 29/31 | | 93.54 | 36/44 | 81.81 | | | | | .181 |
| Soft, doughy skin | 59/75 | 78.66 | | | | 24/30 | 80.00 | | 35/45 | | | | 77.77 | .954 | 24/31 | | 77.41 | 35/44 | 79.54 | | | | | .948 |
| Molluscoid pseudotumor | 22/75 | 29.33 | | | | 13/30 | 43.33 | | 9/45 | | | | 20.00 | .055 | 10/31 | | 32.25 | 12/44 | 27.27 | | | | | .834 |
| Subcutaneous spheroids | 18/65 | 27.60 | | | | 8/24 | 33.33 | | 10/41 | | | | 24.39 | .623 | 10/29 | | 34.48 | 8/36 | 22.22 | | | | | .412 |
| Inguinal/umbilical/incisional hernia | 27/75 | 36.00 | | | | 10/30 | 33.33 | | 17/45 | | | | 37.77 | .883 | 18/31 | | 58.06 | 9/44 | 20.45 | | | | | **.002** |
| Piezogenic papules | 65/75 | 86.66 | | | | 24/30 | 80.00 | | 39/45 | | | | 86.66 | .652 | 26/31 | | 83.87 | 39/44 | 88.63 | | | | | .800 |
| Light blue sclerae | 44/75 | 58.66 | | | | 13/30 | 43.33 | | 31/45 | | | | 68.88 | **.049** | 26/31 | | 83.87 | 18/44 | 40.90 | | | | | **.001** |
| Uvula abnormalities | 26/59 | 44.06 | | | | 16/26 | 61.53 | | 10/33 | | | | 30.30 | **.032** | 13/26 | | 50.00 | 13/33 | 39.39 | | | | | .581 |
| Acquired cutis laxa/premature skin aging | 31/75 | 41.33 | | | | 10/30 | 33.33 | | 21/45 | | | | 46.66 | .363 | 8/31 | | 25.80 | 23/44 | 52.27 | | | | | **.040** |
| Striae distensae/rubrae at young age | 30/75 | 40.00 | | | | 7/30 | 23.33 | | 23/45 | | | | 51.11 | **.030** | 4/31 | | 12.90 | 26/44 | 59.09 | | | | | **.001** |
| Keratosis pilaris/hyperkeratosis of extensor surfaces | 23/75 | 30.66 | | | | 6/30 | 20.00 | | 17/45 | | | | 37.77 | .167 | 8/31 | | 25.80 | 15/44 | 34.09 | | | | | .608 |
| Absent/short lingual frenulum | 12/56 | 21.42 | | | | 5/25 | 20.00 | | 7/31 | | | | 22.58 | .925 | 6/23 | | 26.08 | 6/33 | 18.18 | | | | | .705 |
| Resistance to local anesthetic drugs | 6/32 | 18.75 | | | | 2/14 | 14.28 | | 4/18 | | | | 22.22 | .672 | 0/5 | | 0.00 | 6/27 | 22.22 | | | | | .554 |
| Gingival inflammation/recession | 13/73 | 17.80 | | | | 3/28 | 10.71 | | 10/45 | | | | 22.22 | .349 | 3/31 | | 9.67 | 10/42 | 23.80 | | | | | .211 |
| Xerosis/xerophthalmia | 11/66 | 16.66 | | | | 3/25 | 12.00 | | 8/41 | | | | 19.51 | .649 | 0/28 | | 0.00 | 11/38 | 28.94 | | | | | **.001** |
| Livedo reticularis | 5/75 | 6.66 | | | | 1/30 | 3.33 | | 4/45 | | | | 8.88 | .642 | 2/31 | | 6.45 | 3/44 | 6.81 | | | | | 1.00 |
| **Osteoarticular features** | | | | | | | | | | | | | | | | | | | | | | | | |
| Generalized joint hypermobility (BS ≥ 5/9) | 44/75 | | 58.66 | | | 14/30 | | 46.66 | 30/45 | 66.66 | | | | .138 | 19/31 | | 61.29 | 25/44 | | 56.81 | | | | .881 |
| Sprains | 34/75 | | 45.33 | | | 17/30 | | 56.66 | 17/45 | 37.77 | | | | .169 | 8/31 | | 25.80 | 26/44 | | 59.09 | | | | **.008** |
| ***(Continued)*** | | | | | | | | | | | | | | | | | | | | | | | | |
|  | **Total** | | | | | **Males** | | | **Females** | | | | | **p-value^A^** | **Patients <18** | | | **Patients ≥18** | | | | | | **p-value^B^** |
|  | **N/T** | **%** | | | | **N/T** | **%** | | **N/T** | | | | **%** |  | **N/T** | | **%** | **N/T** | **%** | | | | |  |
| Dislocation | 16/75 | | 21.33 | | | 9/30 | | 30.00 | 7/45 | 15.55 | | | | .226 | 1/31 | | 3.22 | 15/44 | | 34.09 | | | | **.001** |
| Subdislocation | 22/75 | | 29.33 | | | 8/30 | | 26.66 | 14/45 | 31.11 | | | | .876 | 6/31 | | 19.35 | 16/44 | | 36.36 | | | | .181 |
| Articular pain | 30/75 | | 40.00 | | | 12/30 | | 40.00 | 18/45 | 40.00 | | | | .809 | 3/31 | | 9.67 | 27.44 | | 61.36 | | | | **.001** |
| Bilateral pes planus | 62/75 | | 82.66 | | | 25/30 | | 83.33 | 37/45 | 82.22 | | | | .851 | 27/31 | | 87.09 | 35/44 | | 79.54 | | | | .588 |
| Fixed dislocations | 5/75 | | 6.66 | | | 3/30 | | 10.00 | 2/45 | 4.44 | | | | .636 | 1/31 | | 3.22 | 4/44 | | 9.09 | | | | .396 |
| Recurrent inflammatory soft-tissue lesions | 25/74 | | 33.78 | | | 10/30 | | 33.33 | 15/44 | 34.09 | | | | .855 | 4/31 | | 12.90 | 21/43 | | 48.83 | | | | **.002** |
| Temporomandibular joint dysfunction | 21/73 | | 28.76 | | | 6/29 | | 20.68 | 15/44 | 34.09 | | | | .330 | 6/30 | | 20.00 | 15/43 | | 34.88 | | | | .263 |
| Walking difficulties | 17/75 | | 22.66 | | | 8/30 | | 26.66 | 9/45 | 20.00 | | | | .693 | 5/31 | | 16.12 | 12/44 | | 27.27 | | | | .392 |
| Limited walking autonomy | 8/75 | | 10.66 | | | 4/30 | | 13.33 | 4/45 | 8.88 | | | | .818 | 1/31 | | 3.22 | 7/44 | | 15.90 | | | | .129 |
| Congenital hip dysplasia | 2/75 | | 2.66 | | | 1/30 | | 3.33 | 1/45 | 2.22 | | | | 1.00 | 1/31 | | 3.22 | 1/44 | | 2.27 | | | | 1.00 |
| **Orthopedic features** | | | | | | | | | | | | | | | | | | | | | | | | |
| Scoliosis (mild) | 55/74 | | 74.32 | | | 21/30 | | 70.00 | 34/44 | 77.27 | | | | .665 | 21/31 | | 67.74 | 34/43 | | 79.06 | | | | .406 |
| Cervical spine curvature anomalies | 18/68 | | 26.47 | | | 7/28 | | 25.00 | 11/40 | 27.50 | | | | .960 | 3/27 | | 11.11 | 15/41 | | 36.58 | | | | **.040** |
| Dorsal hyperkyphosis | 19/69 | | 27.53 | | | 8/29 | | 27.58 | 11/40 | 27.50 | | | | .790 | 8/28 | | 28.57 | 11/41 | | 26.82 | | | | .908 |
| Lumbar hyperlordosis/hypolordosis | 40/69 | | 57.97 | | | 13/29 | | 44.82 | 27/40 | 67.50 | | | | .101 | 14/28 | | 50.00 | 26/41 | | 63.41 | | | | .389 |
| Minor asymmetry at lower limbs and other body areas | 61/73 | | 83.56 | | | 22/29 | | 75.86 | 38/44 | 86.36 | | | | .636 | 24/30 | | 80.00 | 37/43 | | 86.04 | | | | .715 |
| Disc hernias/protrusion | 13/72 | | 18.05 | | | 4/28 | | 14.28 | 9/44 | 20.45 | | | | .727 | 1/29 | | 3.44 | 12/43 | | 27.90 | | | | **.010** |
| Spondylolisthesis | 9/74 | | 12.16 | | | 3/29 | | 10.34 | 6/45 | 13.33 | | | | .984 | 0/30 | | 0.00 | 9/44 | | 20.45 | | | | **.008** |
| Back pain | 29/75 | | 38.66 | | | 12/30 | | 40.00 | 17/45 | 37.77 | | | | .961 | 4/31 | | 12.90 | 25/44 | | 56.81 | | | | **.001** |
| Cubita/genua valga/halluces valgi | 40/75 | | 53.33 | | | 14/30 | | 46.66 | 26/45 | 57.77 | | | | .478 | 15/31 | | 48.38 | 25/44 | | 56.81 | | | | .627 |
| High arched/narrow palate | 31/62 | | 50.00 | | | 14/28 | | 50.00 | 17/34 | 50.00 | | | | .798 | 16/26 | | 61.53 | 15/36 | | 41.66 | | | | .198 |
| Non-surgical pectus excavatum/carinatum | 17/75 | | 22.66 | | | 12/30 | | 40.00 | 5/45 | 11.11 | | | | **.008** | 6/31 | | 19.35 | 11/44 | | 25.00 | | | | .768 |
| Osteopenia | 11/31 | | 35.48 | | | 3/10 | | 30.00 | 8/21 | 38.09 | | | | .969 | 0/10 | | 0.00 | 11/21 | | 52.38 | | | | **.004** |
| Osteoporosis | 5/31 | | 16.12 | | | 1/10 | | 10.00 | 4/21 | 19.04 | | | | 1.00 | 0/10 | | 0.00 | 5/21 | | 23.80 | | | | .147 |
| Arachnodactyly | 5/75 | | 6.66 | | | 3/30 | | 10.00 | 2/45 | 4.44 | | | | .636 | 4/31 | | 12.90 | 1/44 | | 2.27 | | | | .177 |
| Marfanoid habitus | 1/75 | | 1.33 | | | 1/30 | | 3.33 | 0/45 | 0.00 | | | | .400 | 0/31 | | 0.00 | 1/44 | | 2.27 | | | | 1.00 |
| **Muscular features** | | | | | | | | | | | | | | | | | | | | | | | | |
| Recurrent myalgias/cramps | 31/75 | | | | 41.33 | 15/30 | 50.00 | | 16/45 | | | 35.55 | | .314 | 8/31 | 25.80 | | 23/44 | | | | 52.27 | | **.040** |
| Hypotonia at birth | 11/75 | | | | 14.66 | 6/30 | 20.00 | | 5/45 | | | 11.11 | | .463 | 5/31 | 16.12 | | 6/44 | | | | 13.63 | | .975 |
| ***(Continued)*** | | | | | | | | | | | | | | | | | | | | | | | | |
|  | **Total** | | | | | **Males** | | | **Females** | | | | | **p-value^A^** | **Patients <18** | | | **Patients ≥18** | | | | | | **p-value^B^** |
|  | **N/T** | **%** | | | | **N/T** | **%** | | **N/T** | | | | **%** |  | **N/T** | | **%** | **N/T** | **%** | | | | |  |
| Muscle hypotonia of mild degree | 5/75 | | | | 6.66 | 2/30 | 6.66 | | 3/45 | | | 6.66 | | .636 | 2/31 | 6.45 | | 3/44 | | | | 6.81 | | 1.00 |
| Involuntary muscle contractions | 4/75 | | | | 5.33 | 3/30 | 10.00 | | 1/45 | | | 2.22 | | .345 | 3/31 | 9.67 | | 1/44 | | | | 2.27 | | .376 |
| Fibromyalgia | 1/75 | | | | 1.33 | 0/30 | 0.00 | | 1/45 | | | 2.22 | | 1.00* | 0/31 | 0.00 | | 1/44 | | | | 2.27 | | 1.00 |
| **Gastrointestinal features** | | | | | | | | | | | | | | | | | | | | | | | | |
| Gastroesophageal reflux | 30/75 | | | 40.00 | | 14/30 | 46.66 | | 16/45 | | 35.55 | | | .470 | 7/31 | | 22.58 | 23/44 | | | 52.27 | | | **.019** |
| Defecatory dysfunction | 25/75 | | | 33.33 | | 7/30 | 23.33 | | 18/45 | | 40.00 | | | .211 | 6/31 | | 19.35 | 19/44 | | | 43.18 | | | .056 |
| Delayed gastric/bowel/colonic transit | 20/75 | | | 26.66 | | 5/30 | 16.66 | | 25/45 | | 55.55 | | | .182 | 5/31 | | 16.12 | 15/44 | | | 34.09 | | | .142 |
| Various food intolerances | 12/75 | | | 16.00 | | 5/30 | 16.66 | | 7/45 | | 15.55 | | | .847 | 3/31 | | 9.67 | 9/44 | | | 20.45 | | | .350 |
| Unexplained abdominal pain | 12/75 | | | 16.00 | | 4/30 | 13.33 | | 8/45 | | 17.77 | | | .847 | 2/31 | | 6.45 | 10/44 | | | 22.72 | | | .107 |
| Dysphagia | 7/75 | | | 9.33 | | 3/30 | 10.00 | | 4/45 | | 8.88 | | | .807 | 1/31 | | 3.22 | 6/44 | | | 13.63 | | | .228 |
| Hiatal hernia | 3/73 | | | 4.10 | | 2/29 | 6.89 | | 1/44 | | 2.27 | | | .710 | 0/31 | | 0.00 | 3/42 | | | 7.14 | | | .256 |
| Visceroptosis | 2/75 | | | 2.66 | | 0/30 | 0.00 | | 2/45 | | 4.44 | | | .513 | 0/31 | | 0.00 | 2/44 | | | 4.54 | | | .508 |
| Dolichocolon | 1/74 | | | 1.35 | | 0/30 | 0.00 | | 1/44 | | 2.27 | | | 1.00 | 0/31 | | 0.00 | 1/43 | | | 2.32 | | | 1.00 |
| Confirmed celiac disease | 1/74 | | | 1.35 | | 0/30 | 0.00 | | 1/44 | | 2.27 | | | 1.00 | 1/31 | | 3.22 | 0/43 | | | 0.00 | | | .418 |
| Inflammatory bowel disease | 0/75 | | | 0.00 | | 0/30 | 0.00 | | 0/45 | | 0.00 | | | - | 0/31 | | 0.00 | 0/44 | | | 0.00 | | | - |
| **Cardiovascular features** | | | | | | | | | | | | | | | | | | | | | | | | |
| Mitral valve prolapse | 28/66 | | | 42.42 | | 11/27 | 40.74 | | 17/39 | | | | 43.58 | .981 | 6/27 | | 22.22 | 22/39 | | | | | 56.41 | **.012** |
| Capillary fragility/recurrent epistaxis/gingival bleedings | 31/75 | | | 41.33 | | 8/30 | 26.66 | | 23/45 | | | | 51.11 | .061 | 9/31 | | 29.03 | 22/44 | | | | | 50.00 | .114 |
| Valvular regurgitation with mild hemodynamic involvement | 23/66 | | | 34.84 | | 12/27 | 44.44 | | 11/39 | | | | 28.20 | .271 | 7/27 | | 25.92 | 16/39 | | | | | 41.02 | .315 |
| Varicose veins | 15/75 | | | 20.00 | | 8/30 | 26.66 | | 7/45 | | | | 15.55 | .376 | 1/31 | | 3.22 | 14/44 | | | | | 31.81 | **.002** |
| Raynaud’s phenomenon/acrocyanosis/livedo reticularis | 8/75 | | | 10.66 | | 2/30 | 6.66 | | 6/45 | | | | 13.33 | .593 | 3/31 | | 9.67 | 5/44 | | | | | 11.36 | .883 |
| Low progressive aortic root dilatation | 6/66 | | | 9.09 | | 5/27 | 18.51 | | 1/39 | | | | 2.56 | .074 | 0/27 | | 0.00 | 6/39 | | | | | 15.38 | .073 |
| Aortic ectasia | 2/66 | | | 3.03 | | 2/27 | 7.40 | | 0/39 | | | | 0.00 | .163 | 0/27 | | 0.00 | 2/39 | | | | | 5.12 | .509 |
| **Neuropsychiatric features** | | | | | | | | | | | | | | | | | | | | | | | | |
| Headache/migraine | 31/75 | 41.33 | | | | 9/30 | 30.00 | | 22/45 | | | | 48.88 | .165 | 9/31 | | 29.03 | 22/44 | 50.00 | | | | | .114 |
| Clumsiness | 27/74 | 36.48 | | | | 12/29 | 41.37 | | 15/45 | | | | 33.33 | .649 | 13/31 | | 41.93 | 14/43 | 32.55 | | | | | .560 |
| Chronic fatigue | 27/75 | 36.00 | | | | 14/30 | 46.66 | | 13/45 | | | | 28.88 | .184 | 7/31 | | 22.58 | 20/44 | 45.45 | | | | | .073 |
| Impaired memory/concentration | 23/74 | 31.08 | | | | 8/29 | 27.58 | | 15/45 | | | | 33.33 | .791 | 4/30 | | 13.33 | 19/44 | 43.18 | | | | | **.013** |
| Paresthesia | 19/75 | 25.33 | | | | 9/30 | 30.00 | | 10/45 | | | | 22.22 | .625 | 3/31 | | 9.67 | 16/44 | 36.36 | | | | | **.018** |
| ***(Continued)*** | | | | | | | | | | | | | | | | | | | | | | | | |
|  | **Total** | | | | | **Males** | | | **Females** | | | | | **p-value^A^** | **Patients <18** | | | **Patients ≥18** | | | | | | **p-value^B^** |
|  | **N/T** | **%** | | | | **N/T** | **%** | | **N/T** | | | | **%** |  | **N/T** | | **%** | **N/T** | **%** | | | | |  |
| Cardiovascular dysautonomia | 12/75 | 16.00 | | | | 2/30 | 6.66 | | 10/45 | | | | 22.22 | .139 | 2/31 | | 6.45 | 10/44 | 22.72 | | | | | .107 |
| Allodynia | 10/74 | 13.51 | | | | 2/29 | 6.89 | | 8/45 | | | | 17.77 | .323 | 1/30 | | 3.33 | 9/44 | 20.45 | | | | | **.041** |
| Delayed motor development | 8/75 | 10.66 | | | | 4/30 | 13.33 | | 4/45 | | | | 8.88 | .818 | 4/31 | | 12.90 | 4/44 | 9.09 | | | | | .883 |
| Neuropathic pain | 1/73 | 1.36 | | | | 0/29 | 0.00 | | 1/44 | | | | 2.27 | 1.00 | 0/30 | | 0.00 | 1/43 | 2.32 | | | | | 1.00 |
| Anxiety/panic/fears | 11/75 | 14.66 | | | | 5/30 | 16.66 | | 6/45 | | | | 13.33 | .946 | 3/31 | | 9.67 | 8/44 | 18.18 | | | | | .487 |
| Sleep disturbances | 11/75 | 14.66 | | | | 4/30 | 13.33 | | 7/45 | | | | 15.55 | .946 | 3/31 | | 9.67 | 8/44 | 18.18 | | | | | .487 |
| Depression | 3/75 | 4.00 | | | | 1/30 | 3.33 | | 2/45 | | | | 4.44 | 1.00 | 2/31 | | 6.45 | 1/44 | 2.27 | | | | | .565 |
| **Painkillers** | | | | | | | | | | | | | | | | | | | | | | | | |
| NSAIDs/paracetamol | 37/75 | 49.33 | | | | 14/30 | 46.66 | | 23/45 | | | | 51.11 | .887 | 5/31 | | 16.12 | 32/44 | 72.72 | | | | | **.001** |
| Opioids | 5/75 | 6.66 | | | | 0/30 | 0.00 | | 5/45 | | | | 11.11 | .079 | 1/31 | | 3.22 | 4/44 | 9.09 | | | | | .396 |
| Steroids | 3/75 | 4.00 | | | | 1/30 | 3.33 | | 2/45 | | | | 4.44 | 1.00 | 1/31 | | 3.22 | 2/44 | 4.54 | | | | | 1.00 |
| Antidepressants | 2/75 | 2.66 | | | | 1/30 | 3.33 | | 1/45 | | | | 2.22 | 1.00 | 2/31 | | 6.45 | 0/44 | 0.00 | | | | | .167 |
| Non-pharmacological pain therapy | 9/75 | 12.00 | | | | 4/30 | 13.33 | | 5/45 | | | | 11.11 | .942 | 2/31 | | 6.45 | 7/44 | 15.90 | | | | | .291 |
| **Uro-gynecological features** | | | | | | | | | | | | | | | | | | | | | | | | |
| Meno/metrorrhagias |  |  | | | |  |  | | 5/31 | | | | 16.12 | NV | 0/5 | | 0.00 | 5/26 | 19.23 | | | | | .560 |
| Disabling dysmenorrhea |  |  | | | |  |  | | 7/31 | | | | 22.58 | NV | 0/5 | | 0.00 | 7/26 | 26.92 | | | | | .562 |
| Post-partum hemorrhage |  |  | | | |  |  | | 2/20 | | | | 10.00 | NV | 0/2 | | 0.00 | 2/18 | 11.11 | | | | | 1.00 |
| Urinary stress incontinence | 2/74 | 2.70 | | | | 1/29 | 3.44 | | 1/45 | | | | 2.22 | 1.00 | 0/31 | | 0.00 | 2/43 | 4.65 | | | | | .506 |
| Pelvic prolapse | 1/75 | 1.33 | | | | 0/30 | 0.00 | | 1/45 | | | | 2.22 | 1.00 | 0/31 | | 0.00 | 1/44 | 2.27 | | | | | 1.00 |
| **Atopic features** | | | | | | | | | | | | | | | | | | | | | | | | |
| Allergy/atopy | 24/75 | 32.00 | | | | 13/30 | 43.33 | | 11/45 | | | | 24.44 | .142 | 8/31 | | 25.80 | 16/44 | 36.36 | | | | | .475 |
| Rhinitis/rhinoconjunctivitis | 11/75 | 14.66 | | | | 7/30 | 23.33 | | 4/45 | | | | 8.88 | .161 | 3/31 | | 9.67 | 8/44 | 18.18 | | | | | .487 |
| Confirmed atopic dermatitis | 8/75 | 10.66 | | | | 3/30 | 10.00 | | 5/45 | | | | 11.11 | .818 | 3/31 | | 9.67 | 5/44 | 11.36 | | | | | .883 |
| Asthma | 3/75 | 4.00 | | | | 1/30 | 3.33 | | 2/45 | | | | 4.44 | 1.00 | 1/31 | | 3.22 | 2/44 | 4.54 | | | | | 1.00 |
| **Ocular features** |  |  | | | |  |  | |  | | | |  |  |  | |  |  |  | | | | |  |
| Palpebral ptosis | 24/75 | 32.00 | | | | 9/30 | 30.00 | | 15/45 | | | | 33.33 | .959 | 13/31 | | 41.93 | 11/44 | 25.00 | | | | | .194 |
| Sunken eyes | 24/75 | 32.00 | | | | 12/30 | 40.00 | | 17/45 | | | | 37.77 | .961 | 11/31 | | 35.48 | 18/44 | 40.90 | | | | | .814 |
| Infraorbital creases | 22/75 | 29.33 | | | | 9/30 | 30.00 | | 13/45 | | | | 28.88 | .876 | 13/31 | | 41.93 | 9/44 | 20.45 | | | | | .079 |
| Myopia | 19/75 | 25.33 | | | | 9/30 | 30.00 | | 10/45 | | | | 22.22 | .625 | 2/31 | | 9.52 | 17/44 | 38.63 | | | | | **.002** |
| ***(Continued)*** | | | | | | | | | | | | | | | | | | | | | | | | |
|  | **Total** | | | | | **Males** | | | **Females** | | | | | **p-value^A^** | **Patients <18** | | | **Patients ≥18** | | | | | | **p-value^B^** |
|  | **N/T** | **%** | | | | **N/T** | **%** | | **N/T** | | | | **%** |  | **N/T** | | **%** | **N/T** | **%** | | | | |  |
| Epicanthal fold | 14/75 | 18.66 | | | | 6/30 | 20.00 | | 8/45 | | | | 17.77 | .951 | 10/31 | | 32.25 | 4/44 | 9.09 | | | | | **.025** |
| Strabismus | 6/75 | 8.00 | | | | 4/30 | 13.33 | | 2/45 | | | | 4.44 | .339 | 3/31 | | 9.67 | 3/44 | 6.81 | | | | | .986 |
| Hypertelorism/hypertelorism | 6/75 | 8.00 | | | | 3/30 | 10.00 | | 3/45 | | | | 6.66 | .930 | 5/31 | | 16.12 | 1/44 | 2.27 | | | | | .080 |
| Xerophthalmia | 5/70 | 7.14 | | | | 1/27 | 3.70 | | 4/43 | | | | 9.30 | .642 | 0/30 | | 0.00 | 5/40 | 12.50 | | | | | .066 |
| **Non-ocular features** | | | | | | | | | | | | | | | | | | | | | | | | |
| Micrognathia/retrognathia | 22/75 | 29.33 | | | | 10/30 | 33.33 | | 12/45 | | | | 26.66 | .717 | 13/31 | | 41.93 | 9/44 | 20.45 | | | | | .079 |
| Hypoplastic auricular lobe | 15/75 | 20.00 | | | | 6/30 | 20.00 | | 9/45 | | | | 20.00 | .768 | 12/31 | | 38.70 | 3/44 | 6.81 | | | | | **.001** |
| Anteverted nostrils | 6/75 | 8.00 | | | | 4/30 | 13.33 | | 2/45 | | | | 4.44 | .339 | 3/31 | | 9.67 | 3/44 | 6.81 | | | | | .986 |
| Low-set ears | 4/75 | 5.33 | | | | 3/30 | 10.00 | | 1/45 | | | | 2.22 | .345 | 2/31 | | 9.52 | 2/44 | 4.54 | | | | | 1.00 |
| Elongated philtrum | 2/75 | 2.66 | | | | 2/30 | 6.66 | | 0/45 | | | | 0.00 | .305 | 1/31 | | 3.22 | 1/44 | 2.27 | | | | | 1.00 |

N: number of patients presenting the investigated feature; T: total number of patients in whom the feature was investigated. Significant p-values <0.05 are in bold. A: p-values females vs males; B: p-values patients <18 years vs patients ≥18 years.
